# The Precariat Scale (PRESC): a psychometric tool for assessing precarity across mental, occupational, and sociocultural dimensions

**DOI:** 10.3389/fpsyg.2026.1808999

**Published:** 2026-09-01

**Authors:** Ahmet Keser, Müjgan Şahin, Yunus Gokmen

**Affiliations:** 1 Department of Political Science and International Relations, Hasan Kalyoncu University, Gaziantep, Türkiye; 2 Faculty of Communication, Baskent University, Ankara, Türkiye

**Keywords:** general adult population, new dangerous class, precariat, psychometric validity and reliability, scale development, social psychology

## Abstract

**Introduction:**

The *precariat* is an emerging global demographic comprising unemployed, underemployed, and temporarily employed individuals facing severe socio-economic instability. Despite growing theoretical attention, there is a lack of comprehensive empirical measurement tools. This study addresses this gap by developing and validating the Precariat Scale (PRESC)—a multidimensional instrument designed to assess precarious conditions across three core dimensions: *Sociocultural-Economic* (SCE), *Job Insecurity, Uncertainty and Flexible Working* (JIUFW), and *Mental Condition* (MC).

**Methods:**

Following a rigorous six-stage scale development protocol, data were collected from 421 participants. Psychometric analyses were conducted on a final sample of 394 participants to evaluate the construct validity, factor structure, and reliability of the scale using polychoric correlation matrices.

**Results:**

Psychometric evaluations supported a robust, stable three-factor model corresponding to the hypothesized dimensions (SCE, JIUFW, and MC). The scale demonstrated strong internal consistency, construct validity, and overall psychometric reliability.

**Discussion:**

The PRESC provides a pioneering, empirically validated framework for operationalizing and measuring the multidimensional realities of the precariat. This tool enables researchers and policymakers to quantitatively assess precariousness and investigate its wider socio-economic and psychological implications.

## 1 Introduction

A book authored by Standing (2011) entitled *The precariat: the new dangerous class* triggered new discussions on a new emerging class of unemployed, underemployed, or temporarily employed labor power on a global scale. As reviewed by Allen and Ainley (2011) and Standing (2011) does not remain within the ranks of Marx’s proletariat; instead, he defines a new class including large numbers of people who have been pushed into a new and insecure class, named as “precariat” by the author. The labor power within this new class is composed of an insecure workforce lacking adequate incomes without a “secure work-based identity.” Since then, many discussions have been conducted within the current literature on the topic, as can be seen within the Theoretical and Conceptual Frame section, but unfortunately, *a scale that will provide an opportunity to research the precariat class has not been developed yet*. In other words, although the topic has attracted great attention among academics, *there is still a gap in the literature regarding the development of a statistically valid and reliable scale to research the precariat*.

Within the above-mentioned frame, the main purpose of this study is to develop a valid and reliable Precariat Scale by collecting data from a statistically sufficient sample size, including both women and men participants. This study reviews the literature on the precariat and briefly provides the theoretical and conceptual background in Section 2. Section 3 defines the scale and then discusses the procedures used to develop the scale. Section 4 explains the data collection and methods employed; Section 5 presents the results of scale validity and reliability. Finally, Section 6 briefly discusses the results and concludes.

## 2 Theoretical and conceptual background

As briefly mentioned in the Introduction, with the publication of Standing’s (2011) book “*The Precariat*,” new debates emerged. According to the author, “The globalization era has resulted in a fragmentation of national class structures” (Standing, 2011). Moreover, “Marx’s proletariat, who make up one half of Standing’s portmanteau precarious-proletariat, are now more numerous than ever as worldwide other intermediate classes and the peasantry have collapsed into them or will soon do so.” (Allen and Ainley, 2011). Standing, formally follows the arguments developed by Gorz (1982) who asserted that “changes in the production process had produced a “non-class” encompassing “all those who have been *expelled from production* … or whose capacities are *under-employed* as a result of the automation and computerization of intellectual work.”

Following the Standing (2011, 2014), three new and nuanced approaches and groups superseded (Palęcka and Płucienniczak, 2017, 2020). (1) *Macro sociological focus:* These researchers focused on the economy and the quality of jobs, the labor market, and precarious employment (Olsthoorn, 2014; Mrozowicki and Maciejewska, 2016). (2) *Meso-sociological:* This second group of academics focused on social movements and contentious politics, and the collective identity of the precariat (della Porta et al., 2015). (3) *Individual experiences of precariousness*: The third group focused on the feelings of insecurity, subjugation, fear, and anxiety (Lorey, 2015; Palęcka and Płucienniczak, 2020), and psychology (Rua et al., 2023).

When the emergence and first usage of the word was investigated, it is possible to see that the phrase *precariat* was initially used by French sociologists in the 1980s to describe *temporary or seasonal workers*. When the phrase traveled to Italy, its meaning was enriched, and *precariat* began to imply a precarious existence as a normal state of living, extending its meaning beyond that of French sociologists. In contrast, in Germany, the term *precariat* has been used to describe both temporary workers and the jobless without any hope of social integration. Although the German usage brings the content of the term *precariat* close to Marx’s idea of a *lumpenproletariat*, it is not that either (Allen and Ainley, 2011). For Standing (2011), although far from being homogeneous, the precariat is a “class in the making, increasingly able to identify what it wishes to combat and what it wishes to construct.” According to Paul (2018), the term *precariat* is a portmanteau word made up of *precarious* and *proletariat*, and the phrase refers to those “whose employment and income are unstable, short-term and usually seriously reduced, in contrast to those who have a more permanent status as workers with regular pay and conditions.”

To place the precariat within the existing social classes of the world, it is better to first broadly describe the existing classes. Within the existing classes in different parts of the world, (1) the first one at the uppermost level is an “*elite*,” which consists of a very small number of extraordinarily rich global citizens enjoying all the gifts of the universe. (2) The second upper class is the “*salariat*,” just below the *elite*, who also still enjoy stable and full-time employment. The salariat is trying and hoping to be promoted into the elite. This class enjoys some trappings, such as a good pension, paid holidays, and enterprise benefits. (3) The third class “*proficiens*” is a term produced by combining the traditional phrases of “professional” and “technician,” and consists of a smaller group alongside the salariat. (4) The fourth class is the old “*working class*,” which is below the *proficiens* in terms of income, and consists mostly of the manual employees. According to Allen and Ainley (2011), the growing new class “precariat” comes under these four groups, consisting of a growing crowd of “unemployed and a detached group of socially ill misfits, and living off the dregs of society.”

According to Standing (2011), the members of the new class precariat are not able to completely or partially enjoying the seven forms of labor-related security that social democrats and trade unionists demanded: (1) an adequate income, (2) protection against arbitrary dismissal, (3) job security with barriers to skill dilution, (4) work security protecting against accidents and illness at work, (5) opportunity to gain skills through apprenticeship and training, (6) assurance of an adequate stable income, and (7) representation of their collective rights through independent trades unions with the right to strike. Thus, the members of this class can see no better future for themselves.

In a historical glance, following the new right ideologies and the transition of public administration to a new public management system, almost all paradigms related to governance and employment systems have been changed. With the rise of this new era, trying to govern public systems also according to private-sector methods first ended the Fordist–Keynesian compromise and welfare state retrenchment. These attempts were followed by the degradation of employment, privatization, and contracting, and job tenure, employment security, and employee rights were weakened. As a result, real wages have fallen, and collective protections for the working class were eroded (Wacquant, 2009). Ishchuk et al. (2023) like the emergence of the precariat to the impacts of globalization. The authors argue that globalization has led to “various forms of labor relations flexibility, psychological distress, and a disruption of work culture.” It caused some consequences that “extend to shadow economy dynamics, migration issues, reduced wages, and a decline in taxable income” (Ishchuk et al., 2023). New employer strategies such as “externalization of the workforce and its costs—a strategy encouraged by public authorities and powerfully reinforced by the active marketing of temporary employment agencies” (Wacquant, 2009) have accelerated, and the accelerated, and state structures have handed almost all their production activities over to private contractors. All these developments have changed the structure of the workforce and paved the way for the new class of precariat. For Standing (2011), the precariat is composed of youth, migrants, criminals, and disabled minorities.

Fletcher (2019) aligns the emergence of the precariat with the post-industrial period. He argues that this class is characterized by “social insecurity to which the state’s response has been to secure habituation to insecure labor.” Regarding the characteristics of the precariat, Standing (2014) asserts in an interview that the precariat is characterized by the following:

- Living with unstable labor: Millions of people are being forced to live through unstable jobs.
- Chronic economic insecurity: Millions of people are being forced to live with fluctuating incomes and periodic unemployment.
- The precariat faces chronic uncertainty: This uncertainty is stressful and bad for health. In an open market economy system with flexible labor relations, the only way to provide the precariat with basic economic security is through a guaranteed, unconditional basic income.
- Lack of basic security: Without basic security, people cannot be expected to be responsible citizens or human beings. We become desperate, anomic, alienated, and angry (Standing and Jandric, 2015).

Braga (2016), in contrast, has some objections regarding the ideas developed by Standing (2011). For example, for Standing (2011), unions and traditional social democratic parties were weakened by the current conditions of capitalist globalization. Thus, the new class, the precariat, could only be well represented through the governance of social and economic agencies. For this purpose, the formulation of public policies should be democratized through the presence of representatives from the precariat. Although Braga (2016) is not against the idea of democratization of the state apparatus, nevertheless, looking at the Brazilian experience, he argues that “it is clear that there is no truly credible reason for the precariat to defend the Universal Basic Income as its priority flag of mobilization.” Briefly, leaving rights in the hands of policymakers may not be the solution. According to Braga (2016), there is no contradiction between the precariat and trade unionism. At least, there is no insurmountable conflict between them in Brazil and Portugal. In each case, there is a reality that “the prevalence of the precariat, ranging from 25 to 40%, with an alarming trend of increase, underscores its emergence as a distinct and influential social class” (Ishchuk et al., 2023). The discussion by Ishchuk et al. (2023), asserting that “the instability of their civil positions leads many members to experience blurred consciousness, resulting in various manifestations such as anomalous behavior and involvement in criminal or delinquent activities,” also indicates the significance of the issue for global society.

Standing (2014) argues that the relationship of the precariat to the labor market is highly opportunistic. Because the current work environment tends to be temporary, it enables a nomadic pattern of drifting. The rise of precarious forms of employment provided employers with an opportunity to manage uneven labor demand. The precariat is increasingly denied rights accorded to previous generations, and all the risks of labor underutilization have been placed on workers who can be hired and fired.

Of course, the *precarity* cannot and should not be evaluated as “just an economic phenomenon that affects the conditions and pay of both skilled and unskilled workers. The privatization of the economy has also had a detrimental impact on the fabric of society as a whole.” (Paul, 2018). Furthermore, the current literature shows that there are also differences within the interpretation of the characteristics or criteria to be included in the precariat from country to country. For example, Fishman (2022) argues that “the Russian interpretation of the precariat differs markedly from the Western one.” According to the author, this finding is relevant “to both the composition of the precariat and its place in the social structure.” In the Russian interpretation, the precariat includes almost half of the society, and almost coincides with the Russian middle class. In other words, as an emerging social class, the precariat has started challenging the established norms of economic life. The social structure of modern society has undergone constant changes, and the concept of precariat has gained prominence in scientific discourse, for example, in Türkiye, at least 58 dissertations on the precariat were conducted at 35 universities between 2013 and 2024 (Kıroğlu and Ataklı Yavuz, 2024, p. 6). At this point, to compare the cases of Russia and Türkiye, it is considered appropriate to draw on the three groups that Standing identifies as constituting the precariat. According to Standing, the precariat comprises three broad groups. The first consists of individuals from working-class families or social groups who have experienced downward class mobility. The second includes highly educated individuals who, despite their educational qualifications, are unable to secure stable and protected employment, as well as salaried workers who are concerned that their children may become part of the precariat. The third group comprises migrants and ethnic minorities (Standing, 2014, p. 11).

The circumstances of the first group may exhibit certain similarities between Türkiye, which has pursued neoliberal economic policies particularly since the 1980s, and Russia, which underwent a major economic restructuring following the glasnost and perestroika reforms and the transformations of the post-1990 period. However, a notable difference emerges for the second group, particularly in terms of the demographic weight of the younger population. Young people aged 15–24 account for 15% of Türkiye’s population (UNFPA in Türkiye, 2024), whereas the same age group represents 9.54% of the total population in Russia (Populationpyramids, 2025). That is why Türkiye has to develop more efficient policies to employ and satisfy the needs of younger generations.

A further significant difference emerges in relation to the third group. Following the outbreak of the Syrian conflict in 2011, Türkiye became a major destination for large-scale migration from Syria. The Syrian migrant population in Türkiye reached approximately 3.6 million at its peak and, although this figure has declined to around 2.5 million following the regime change in Syria, a substantial migrant population continues to reside in the country. This demographic context creates an important distinction between the two countries in terms of the challenges associated with migrants as a component of the precariat. Accordingly, examining the conditions of these groups across different national contexts and identifying similarities and differences between them requires the development of a Precariat Scale whose reliability and validity have been systematically tested.

As explained above, although the concept has gained significant attention both from academics and policymakers, “the precariat remains inadequately defined, with various interpretations and debates surrounding its characterization” (Ishchuk et al., 2023), and currently, there is no standardized scale to measure the status of the people within the precariat.

Although a comprehensive instrument specifically designed to measure the multidimensional condition of the precariat remains limited, several existing scales assess related constructs. For example, the Employment Precariousness Scale (EPRES) evaluates precarious employment through dimensions such as temporariness, disempowerment, vulnerability, wages, rights, and the exercise of workplace rights (Vives et al., 2010), while the Job Insecurity Scale (JIS) primarily focuses on individuals’ perceived threat of job loss and uncertainty regarding employment continuity (De Witte, 2000; Vander Elst et al., 2014). Other instruments address related psychosocial consequences, including economic insecurity, psychological distress, burnout, and social well-being, but generally examine these dimensions as separate constructs. Although these measures provide valuable insights into specific aspects of precariousness, they do not fully capture the broader interaction between employment insecurity, sociocultural-economic conditions, and mental well-being that characterizes the precariat as conceptualized by Standing. PRESC therefore differs from related instruments by integrating these interconnected dimensions within a single multidimensional framework.

In recent research, the dimensions and indicators of precarious work are provided in an index by Lee and Baek (2025) as follows:

1. Dimension 1: employment instability; indicators: (a) contractual relationship insecurity and (b) work insecurity.
2. Dimension 2: income inadequacy; indicator: low income.
3. Dimension 3: lack of social protection; Indicator: low coverage of social insurance.

Thus, this study aims to fill this gap by collecting data from a sufficient number of participants as a sample group and developing a valid and reliable Precariat Scale.

## 3 Development of the scale

### 3.1 Definition of the scale

Using the ideas discussed in the preceding part, we improved the Precariat Scale (PRESC). We specified three factors [sociocultural-economic (SCE), job insecurity, uncertainty and flexible working (JIUFW), and mental condition (MC)] to release people’s precarious structures in the PRESC.

### 3.2 Procedures

We used the integrated six-phase improvement process suggested by Kyriazos and Stalikas (2018) and DeVellis and Thorpe (2022) to enhance the PRESC. Below is a summary of the procedure’s steps.

#### 3.2.1 Creating an initial pool of items

Creating an item pool may initially make some items seem negative. In contrast, superfluous items might separate and remain outside the scale, while fully scaled items will move closer to one another. Therefore, it is important to maintain a wide item pool and create items that allow the scale to be completely portrayed, as stated by DeVellis and Thorpe (2022). Following the recommendations of DeVellis and Thorpe (2022), we prioritized creating a comprehensive initial item pool to fully capture the conceptual domain of the scale. To provide an adequate buffer against item loss during the psychometric validation phases and to obtain a final, simpler scale, an item pool of 20 expressions was used to improve the PRESC for this investigation. The content for these 20 expressions was generated deductively based on an extensive review of the existing literature. During the drafting phase, great care was taken to ensure semantic clarity by creating statements that represented a single judgment, avoiding ambiguous questions, and strictly adhering to the grammar and spelling rules of Turkish language.

#### 3.2.2 Determining the measurement format

After reaching agreement on the question pool, we used a 5-point Likert scale to rate each item, with 1 denoting “*strongly disagree*” and 5 “*strongly agree*” in our survey questionnaire.

#### 3.2.3 Examining the item pool

To establish Face and Content Validity, the initial 20-item pool underwent rigorous screening by an expert panel. The panel included eight experts: four academicians specializing in Turkish language and literature to evaluate grammatical structure and syntactic clarity, and four subject matter experts to assess the items’ theoretical relevance to the construct. The experts were asked to evaluate the pool by rating each item on a 4-point scale from 1 (not relevant) to 4 (highly relevant). Based on the panel’s feedback and consensus, we implemented specific reduction and refinement procedures. While 3 items (items 4, 10, and 18) were entirely removed from the scale because the experts deemed them redundant or outside the scope of the target construct, a total of 4 items were reworded (items 3, 7, 11, and 15) to improve their intelligibility and grammatical structure.

#### 3.2.4 Pilot study

A larger pilot study is far more successful at this stage than using inflated estimations to avoid imprecision. Expanding the pilot study’s sample size to 60–100 individuals may provide far more accurate information on crucial topics (Teare et al., 2014). For our investigation, we thus polled a sample of 78 participants. To examine small-scale validity and reliability assessments, which offer essential adjustments for the scale, the data gathered from the pilot research were subjected to item analysis (17 items). Since we have ordered categorical data, we used polychoric correlation matrices, which are more appropriate to recover the true factor model than Pearson correlations (Holgado-Tello et al., 2010; Lee et al., 2012; Flora et al., 2012; Baglin, 2014; Barendse et al., 2015; Watkins, 2018; Kiwanuka et al., 2022). Table A1 (Appendix A in supplementary file) shows the ordinal alpha if-item-deleted and the ordinal corrected item-total correlation computed by the “*alpha*” function in the “*psych*” R package based on a polychoric correlation matrix. Table A1 (Appendix A in supplementary file) shows that some items (PRESC9 and PRESC20) have ordinal corrected item-total correlations that fall below the 0.2 threshold (Jonsson, 2000; Streiner et al., 2003). The ordinal alpha if-ordinal-item-deleted expresses the Cronbach’s alpha reliability coefficient for the internal consistency of the scale when the relevant item is eliminated from the scale (Mitchell and Bradley, 2001). In light of the findings of Table A1 (Appendix A in supplementary file), the scale’s Cronbach’s alpha score rises when PRESC9 and PRESC20 items with ordinal corrected item-total correlations under the 0.2 threshold are eliminated. Thus, *PRESC9 and PRESC20 items were eliminated from the PRESC*. As a result, the scale’s 15 items meet the criteria for item analysis.

#### 3.2.5 Assessing items

After the item pool has been developed, examined, and applied to the sample study as previously expressed, the next step is to examine and evaluate each item independently. Together with item development, this stage might be referred to as the heart of scale development. Item reliability indicates the scale’s internal consistency, and a strong correlation between the items supports this consistency. Exploratory Factor Analysis (EFA) and Correlation Analysis (CA) were employed based on polychoric correlation matrices to assess the PRESC’s validity and reliability in the pilot study. We conducted EFA using the Promax oblique technique that executes faster than Oblimin (Basto and Pereira, 2012) and yields virtually identical structural conclusions with Direct Oblimin and Geomin, and is significantly more suitable than orthogonal methods (Basto and Pereira, 2012; Watts et al., 2023) using the “*fa*” function in the “*psych*” R package. Inter-item polychoric correlations were computed using the “*lavCor*” function in the “*lavaan*” R package. To determine the number of factors, we applied parallel analysis and identified the optimal factor number as 3. Table A2 (Appendix A in supplementary file) displays the outcomes of EFA and CA. The root mean square of the residuals (RMSRs) is 0.04967 < 0.05, indicating a good model fit and referring the unexplained variance in the item correlations is negligible (Hu and Bentler, 1999). A well-defined structure is also shown by factor analysis when loadings should more than 0.70 (Hair et al., 2014), and the ratio (the rate of the square loadings of the larger loading to the smaller loading for each variable), which is obtained from a simple three-step process and used for identifying the significant loadings and to detect potential cross-loading for each variable, should be above 2.0 (Hair et al., 2019). Almost all factor loadings [bold values in Table A2 (Appendix A in supplementary file)] of the three factors [SCE (the 1st, 2nd, 3rd, 5th, 6th, 7th, 8th items), JIUFW (from the 11th–15th items), and MC (the 16th, 17th, and 19th items)] are bigger than the limit of 0.70. Additionally, all ratios exceed the limit of 2.0, indicating negligible cross-loading for each variable. Furthermore, factor analysis may be regarded as acceptable as it supports a well-defined structure and examines the findings, since inter-item polychoric correlations of the factors in the scale are bigger than 0.30 (Hair et al., 2014). All findings indicate that factor analysis is suitable and further support an unambiguous structure.

#### 3.2.6 Optimizing scale length

The number of items and their relationship impact a scale’s Cronbach’s alpha scores. Smaller scales may have less reliability, but participants are more likely to reply to them. For this reason, scales must have a well-established ratio of reliability to shortness. When a sample is large enough, it is frequently divided into two subsamples at random. These subsamples are then evaluated by calculating their reliability scores and cross-checked. If subsamples were gathered in the same way, the analytical outcomes should also be comparable. According to DeVellis and Thorpe (2022), this method suggests that items with low comparable ordinal corrected item-total correlation values would be removed from the scale, but those with high comparable ordinal corrected item-total correlation in both subsamples (*n*_1_ and *n*_2_) would be included in the scale. The findings of the ordinal corrected item-total correlations based on polychoric correlation matrices for two subsamples (*n*_1_ and *n*_2_) in the pilot study are displayed in the Table A3 (Appendix A in supplementary file). The results in Table A3 show that the ordinal corrected item-total correlations for the two subsamples are comparable to each other and above the limit value of 0.2 (Streiner et al., 2003). Therefore, prior to the data collection phase, we determined that the scale should include 15 items.

## 4 Data collection and methods

### 4.1 Data collection

#### 4.1.1 Minimum sample size

To obtain more reliable findings for scale development, the sample size should be carefully selected based on the population. The Turkish Statistical Institute (TURKSTAT, 2025) reports that more than 85.7 million people are living in Türkiye, 61.5 million of whom are older than 18 [Supreme Election Board (SEB), 2024]. According to Krejcie and Morgan's (1970) technique, a minimum sample size of 384 was required for this study to represent the population with a 95% confidence interval (CI) and a 5% margin of error.

#### 4.1.2 Sampling

To better reflect the demographics of the population and prevent any sample bias, the survey was conducted using some methodologies. An online questionnaire form that the authors produced was sent to people in Türkiye via random social networks and communication platforms; some forms were also distributed by hand. The questionnaire was filled out by 421 individuals (all over the age of 18) between 15 April 2024 and 25 October 2024, when data gathering concluded.

The sample characteristics, as determined by descriptive statistics, are analyzed and displayed in Table 1 to ensure that the subgroups of gender, marital status, occupation, etc., have a balanced distribution consistent with the population. The final sample comprised 394 adults (after eliminating forms with missing values and which did not meet basic assumptions) aged 18 years and above living in Türkiye. The gender distribution was nearly balanced, with 50.5% women and 49.5% men, while 52.3% of the participants were married and 41.9% were single. Regarding employment status, 38.6% worked in the public sector, 39.8% in the private sector, and 21.6% were self-employed. The sample also included individuals with different levels of social security coverage, with 24.4% reporting no social security. Participants represented a broad age range, although the majority were between 23 and 45 years of age. Educational backgrounds were also diverse, ranging from no formal education to graduate-level qualifications; 42.1% held a bachelor’s degree, and 11.4% had a graduate degree. The inclusion of adults with varying demographic, occupational, educational, and social security backgrounds provided a heterogeneous sample suitable for assessing the multidimensional structure of precarity in the context of Türkiye. Consequently, based on random sampling, it may be concluded that the sample utilized in this study broadly represents the demographics of the population.

#### 4.1.3 Ethical consideration

This study was approved by the Institutional Review Board and Ethics Committee of Hasan Kalyoncu University (HKU) (project number: E-97105791-050.04-62486). Participants in our study were given pertinent information regarding the scale and the purpose of the investigation prior to the application. To obtain their consent, they were assured of anonymity and informed that participation was completely voluntary. Every step of the study was carried out with ethical issues in mind, and participant identity was meticulously preserved.

#### 4.1.4 Analyzing how well the data fits the assumptions

We examined how well the data matched the assumptions using the appropriate statistical methods, such as the absence of missing data, the absence of single and multiple extreme values, the normal distribution, the overall representation of the demographic features of the population, etc.

##### 4.1.4.1 Eliminating missing data

16 missing data items were discovered in the 421 replies to our survey. We had the data from 405 participants after eliminating the missing data.

##### 4.1.4.2 Determining single/multi-variable extreme values

To find single-variable extreme values, we used standard *Z*-scores with an absolute value greater than 3, as stated by Kannan et al. (2015). Nevertheless, Mahalanobis distances were used to adjust for multi-variable extreme values. Data from 394 individuals were finally included in the study after 11 participants’ data were eliminated based on the presumption of single and multi-variable extreme values.

##### 4.1.4.3 Checking normal distribution

Checking ordinal, Likert-type, or categorical responses for continuous multivariate normality is statistically inappropriate because discrete data structurally violate continuous distribution assumptions (Flora and Curran, 2004; Holgado-Tello et al., 2010). Thus, we conducted validity and reliability analyses based on polychoric correlation matrices that do not assume the observed variables are normally distributed (Li, 2015).

##### 4.1.4.4 Examining the general reflection of demographic features of the population

The sample characteristics, as determined by descriptive statistics, are analyzed and displayed in Table 1 to ensure that the subgroups of gender, marital status, occupation, etc., have a balanced distribution consistent with the population. Consequently, based on random sampling, it may be concluded that the sample utilized in this study broadly represents the demographics of the population.

**Table 1:** Sample descriptive statistics of items.

Variable	*f*	%
1. Gender		
Female	199	50.5
Male	195	49.5
2. Marital status		
Married	206	52.3
Single	165	41.9
Widowed	3	0.8
Divorced/separated	20	5.1
3. Occupation		
Public sector	152	38.6
Private sector	157	39.8
Self-employment	85	21.6
4. Assurance status		
Having private social security	19	4.8
Having social security	279	70.8
Not having social security	96	24.4
5. Age		
18–22	36	9.1
23–28	86	21.8
29–33	77	19.5
34–40	65	16.5
41–45	80	20.3
46–50	25	6.3
51–55	9	2.3
56–60	8	2
61–65	3	0.8
≥65	5	1.3
6. Education status		
No education	7	1.8
Primary/secondary school	48	12.2
High school	78	19.8
Associate degree	50	12.7
Bachelor’s degree	166	42.1
Graduate degree	45	11.4

Item ID	Items^a^	X̄	SD
PRESC1	Her yaz tatile giderim *(I go on vacation every summer)*.	2.546	1.221
PRESC2	Haftada en az bir gün arkadaşlarımla vakit geçiririm *(I spend time with my friends at least one day a week)*.	3.008	1.281
PRESC3	Bir hobiye sahibim ve zaman ayırabiliyorum *(I have a hobby and can devote time to it)*.	2.820	1.258
PRESC5	Kazancımı yeterli buluyorum *(I find my income sufficient)*.	2.591	1.195
PRESC6	Temel besin maddelerine ulaşabiliyorum *(I am able to access basic food supplies)*.	3.129	1.228
PRESC7	Yaşadığım evden memnunum ve yeterli alana sahibim *(I am satisfied with my home and have enough space)*.	2.840	1.295
PRESC8	Sağlık giderlerimi karşılayabiliyorum ve sağlık sigortam var *(I can cover my health expenses and have health insurance)*.	2.975	1.327
PRESC11^b^	Yaptığım işte yükselme şansım yok *(There is no chance for promotion in my job)*.	2.805	1.280
PRESC12^b^	Güvencesiz bir işte olmak beni kaygılandırıyor *(Being in insecure employment makes me anxious)*.	2.447	1.295
PRESC13^b^	İşten ayrılırsam kolaylıkla iş bulamam *(If I lose my job, I would have difficulty finding another)*.	2.442	1.285
PRESC14^b^	İşimde dinlenme zamanım neredeyse hiç yok *(I almost never have time to rest at work)*.	2.850	1.217
PRESC15^b^	İş saatlerimin düzensizliğinden dolayı zamanı yönetemiyorum *(I cannot manage my time due to irregular working hours)*.	2.848	1.293
PRESC16^b^	Psikolojik olarak tükenmiş hissediyorum *(I feel psychologically burned out)*.	2.510	1.335
PRESC17^b^	Kendimi yalnız hissediyorum *(I feel lonely)*.	2.832	1.382
PRESC19^b^	Psikolojik bir desteğe ihtiyacım var *(I need psychological support).*	2.888	1.375

*n* = 394.

a The Turkish and English language versions of the scale are expressed in Appendix B.

b Reverse scores are computed.

### 4.2 Methods

We conducted widely used validity and reliability methods. The scale was initially validated using Construct Validity (CONSV), Content Validity (CONTV), Face Validity (FV), Structural Validity [Exploratory Factor Analysis (EFA) and Confirmatory Factor Analysis (CFA)], Convergent Validity Analysis (CVA), and Discriminant Validity Analysis (DVA) methods. The reliability of the scale was then evaluated using Composite Reliability Analysis (CRA) and Internal Consistency Analysis (ICA). Given the ordinal nature of the items, since our data were ordered categorical data (a Likert-type scale), the data were assessed using RStudio (version 2026.05.1 Build 225), which provides extra analysis tools to conduct validity and reliability analyses based on the polychoric correlation matrices, rather than classic statistical programs. We perform all validity and reliability methods based on polychoric correlation matrices using “*lavaan*,” “*psych*,” “*dynamic*,” “*semTools*,” and “*dplyr*” R packages.

#### 4.2.1 Scale validity

As suggested by Arafat et al. (2016), the validity of the constructed PRESC is assessed using CONSV, CONTV, FV, Structural Validity (EFA and CFA), CVA, and DVA.

In Construct Validity (CONSV), the researcher looks at whether the test captures the concept it’s supposed to. In Content Validity (CONTV), how effectively an instrument captures the field of research and the conceptual description of a thought is evaluated by this validity. Face Validity (FV) examines how well an instrument may be understood and relevant to the target population and pertains to a critical assessment of an instrument following its creation, often involving pilot testing.

For Structural Validity, we applied the most commonly used methods, such as EFA and CFA. Given the ordinal nature of the items, measured on a Likert-type scale in our study, we conducted EFA based on polychoric correlation matrices, which are more appropriate for recovering the true factor model than Pearson correlations (Holgado-Tello et al., 2010; Lee et al., 2012; Flora et al., 2012; Baglin, 2014; Barendse et al., 2015; Watkins, 2018; Kiwanuka et al., 2022). We used the Promax oblique technique, which executes faster than Oblimin (Basto and Pereira, 2012) and yields virtually identical structural conclusions to those from Oblimin, and Geomin, and is significantly more suitable than orthogonal methods (Basto and Pereira, 2012; Watts et al., 2023).

We conducted CFA by using the robust Weighted Least Squares Mean and Variance adjusted (WLSMV) estimation, which is suitable for polychoric matrices with an oblique rotation to achieve the most accurate and reproducible solutions (Lloret et al., 2017; Koziol and Bovaird, 2017). McNeish (2023) expressed that the conventional guidelines derived from assuming continuous responses [e.g., Root Mean Square Error of Approximation (RMSEA) < 0.06, CFI and TLI > 0.95] do not generalize to factor models applied to discrete responses (e.g., ordered categorical data in the Likert-type scale), since the discrete responses provide less information, which is about misfit is passed to fit indices, than continuous responses. Thus, conventional guidelines may be too lenient and lose their ability to identify that a model may have a poor fit. The author provided one possible solution by extending the recently developed Dynamic Fit Index (Wolf and McNeish, 2023) to accommodate discrete responses.

Convergent validity (CV) is the extent to which a construct captures variance in its variables. Convergent Validity was evaluated by calculating the average variance extracted (AVE) for each component. A greater AVE value of 0.50 suggests that the construct explains at least 50% of the variation in the indicators, as advised by Hair et al. (2022), whereas the lowest permissible AVE score is 0.50.

Discriminant Validity Analysis (DVA) examines the relationships between the latent variables to ascertain if the scale accurately reflects different components. The Heterotrait-Monotrait Ratio of Correlations (HTMT) value, a novel method suggested by Henseler et al. (2015) and characterized as high performance in classification by Voorhees et al. (2016) and Franke and Sarstedt (2019), was computed to assess the discriminant validity of the scale. An HTMT score greater than 0.90 would suggest a deficiency in discriminant validity. Hair et al. (2022) recommend a lower, more conservative limit score, such as 0.85, where findings are potentially more diverse.

#### 4.2.2 Reliability

The scale’s reliability is assessed through Composite Reliability Analysis (CRA) and Internal Consistency Analysis (ICA) based on polychoric correlation matrices.

In internal consistency analysis (ICA), the scale’s internal consistency is evaluated using ordinal alpha and McDonald’s omega based on polychoric correlation matrices. Ordinal alpha values are expected to exceed 0.7. Moreover, the accepted standard for internal consistency reliability states that all inter-item polychoric correlations should be greater than the minimum level of 0.30 and that the scale’s ordinal corrected item-total correlation values should be greater than the 0.20 acceptable value (Ravichandran and Rai, 1999; Jonsson, 2000; Streiner et al., 2003; Hair et al., 2014). For McDonald’s omega analysis, the thresholds generally mirror the traditional cut-offs used for Cronbach’s alpha (Hayes and Coutts, 2020). Omega total (ω_t_) should be greater than the limit value of 0.7, and all items should exhibit adequate standardized ordinal factor loadings on the primary construct, exceeding the recommended thresholds (>0.45) for practical significance and Structural Validity (Brown, 2015; Tabachnick and Fidell, 2019).

Composite Reliability (CR) scores based on polychoric correlation matrices were also used to determine the internal consistency of a scale’s factors. A factor is deemed internally consistent if its CR omega total (ω_t_) score exceeds the cutoff point of 0.7 (Hayes and Coutts, 2020).

## 5 Results

### 5.1 Results of scale validity

In Construct Validity *(CONSV)*, the experts identified the scale elements that fit the hypothetical structure. The expert declarations guaranteed the Content Validity *(CONTV)* of the developed PRESC. For Face Validity (FV), the experts and participants concluded that all items on the scale were relevant and clear for the target population after reviewing the pilot research and evaluation results.

#### 5.1.1 Structural validity

As advised by Fokkema and Greiff (2017), the sample was divided into two equal subsamples using random sampling to prevent doing exploratory and confirmatory analyses on the same dataset, which raises the possibility of overfitting. Next, two subsamples (*n*_1_ and *n*_2_) were subjected to independent EFA and CFA based on polychoric correlation matrices.

#### 5.1.2 EFA

For first subsample (*n*_1_ = 197), we conducted EFA based on polychoric correlation matrices (Holgado-Tello et al., 2010; Lee et al., 2012; Flora et al., 2012; Baglin, 2014; Barendse et al., 2015; Watkins, 2018; Kiwanuka et al., 2022) with the Promax oblique technique (Basto and Pereira, 2012; Watts et al., 2023) using the “*fa*” function in the “*psych*” R package. To determine the number of factors, we applied parallel analysis and identified the optimal factor number as 3. The EFA findings are shown in Table 2.

**Table 2:** The findings of EFA.

Item identity (ID)	Factors loadings			PVE (%)	TVE (%)
	SCE	JIUFW	MC		
PRESC1	**0.726**	−0.024	−0.08	28.082	63.782
PRESC2	**0.794**	0.044	0.01		
PRESC3	**0.687**	−0.069	0.022		
PRESC5	**0.906**	0.042	0.08		
PRESC6	**0.721**	−0.079	0.026		
PRESC7	**0.812**	0.007	0.011		
PRESC8	**0.725**	0.069	−0.114		
PRESC11*	0.038	**0.690**	−0.019	20.733	
PRESC12*	0.018	**0.837**	−0.05		
PRESC13*	0.048	**0.764**	0.039		
PRESC14*	−0.134	**0.836**	−0.038		
PRESC15*	−0.027	**0.699**	0.086		
PRESC16*	0.076	0.24	**0.747**	14.966	
PRESC17*	−0.051	−0.054	**0.899**		
PRESC19*	−0.032	−0.012	**0.865**		

PVE, proportion variance explained; TVE, total variance explained. *n*_1_ = 194 is randomly chosen from the main sample (*n* = 394). Bold values indicate the dominant factor loadings for each item. *Reverse scores are computed.

The root mean square of the residuals (RMSRs) is 0.03863 < 0.05, indicating a good model fit and suggesting that the unexplained variance in the item correlations is negligible (Hu and Bentler, 1999). Almost all factor loadings (bold font in Table 2) of the three factors [SCE (the 1st, 2nd, 3rd, 5th, 6th, 7th, 8th items), JIUFW (from the 11th–15th items), and MC (16th, 17th, and 19th items)] are bigger than the limit of 0.70 (Hair et al., 2014). Additionally, all ratios (the rate of the square loadings of the larger loading to the smaller loading for each variable) obtained from a simple three-step process are above the limit value of 2.0, indicating ignorable cross-loading for each variable (Hair et al., 2019). Thus, factor analysis may be considered suitable, and these results also show that the scale is structurally valid.

#### 5.1.3 CFA

To confirm the component structure found in the EFA, we performed a CFA on the second subsample (n_2_ = 197) by using the robust WLSMV estimation based on polychoric correlation matrices, which is suitable for polychoric matrices with an oblique rotation to achieve the most accurate and reproducible solutions (Lloret et al., 2017). We obtained all results using the “*cfa*” function in the “*lavaan*” package and the “*cfaHB*” function in the “*dynamic*” R package. Because the robust WLSMV estimation was utilized for ordinal data, model fit was evaluated using Dynamic Fit Index (DFI) cutoffs specifically simulated for this type of model’s parameters (McNeish, 2023). The empirical fit indices (RMSEA = 0, CFI = 1, SRMR = 0.039) successfully meet the stringent Level-1 dynamic thresholds (RMSEA < 0.08, CFI > 0.957, SRMR < 0.047), indicating that the model structure is robust and even minor misspecifications can be confidently rejected. As a result, we deduced that the scale used in our research demonstrated Structural Validity.

Based on polychoric correlation matrices, while in Convergent Validity Analysis (CVA) we computed AVE values of the factors using “*psych*” and “*dplyr*” R packages, in Discriminant Validity Analysis (DVA), we calculated the components’ HTMT scores using the “*htmt*” function in the “*semTools*” R package for the developed PRESC. The outcomes of the CVA and DVA are indicated in Table 3.

**Table 3:** The outcomes of CVA and DVA.

Factor	Item ID	AVE	HTMT		
			SCE	JIUFW	MC
SCE	1, 2, 3, 5, 6, 7, and 8	0.623	–		
JIUFW	11, 12, 13, 14, and 15	0.630	0.488	–	
MC	16, 17, and 19	0.723	0.529	0.644	–

*n* = 394.

The scale’s Convergent Validity is found to be appropriate based on an analysis of the AVE values of each of its components (Table 3), since each AVE value is above the 0.5 threshold recommended by Fornell and Larcker (1981) and Hair et al. (2022). The discriminant validity of the PRESC is further supported by the fact that all of the HTMT scores in Table 3 are below the 0.85/0.90 threshold suggested by Henseler et al. (2015), Franke and Sarstedt (2019), and Hair et al. (2022).

### 5.2 Results of scale reliability

To assess the scale’s reliability, we performed CRA and ICA based on polychoric correlation matrices. Table 4 displays the findings of ICA and CRA for the developed scale. The findings of ICA and CRA were computed using the “*lavCor*” function in the “*lavaan*” package and the “*alpha*” and “*omega*” functions in the “*psych*” R package, respectively.

**Table 4:** The outcomes of ICA and CRA.

Factor	Item ID	ICA^a^																			CRA^a^
		Inter-item polychoric correlations															Cronbach’s alpha		McDonald’s omega		
																	Ordinal corrected item-total correlation	Ordinal alpha	Standardized ordinal factor loading (*λ*)	Omega total (*ωt*)	CR Omega total (*ωt*)
		1	2	3	4	5	6	7	8	9	10	11	12	13	14	15					
SCE	(1) PRESC1	–															0.767	0.922	0.803	0.922	0.923
	(2) PRESC2	0.615	–														0.700		0.729		
	(3) PRESC3	0.610	0.671	–													0.728		0.759		
	(4) PRESC5	0.690	0.584	0.648	–												0.795		0.836		
	(5) PRESC6	0.649	0.573	0.576	0.681	–											0.779		0.819		
	(6) PRESC7	0.656	0.560	0.616	0.711	0.735	–										0.804		0.846		
	(7) PRESC8	0.596	0.523	0.527	0.625	0.654	0.696	–									0.722		0.757		
JIUFW	(8) PRESC11								–								0.680	0.896	0.721	0.897	0.897
	(9) PRESC12								0.594	–							0.770		0.824		
	(10) PRESC13								0.604	0.698	–						0.730		0.778		
	(11) PRESC14								0.607	0.674	0.640	–					0.803		0.866		
	(12) PRESC15								0.544	0.638	0.550	0.774	–				0.735		0.789		
MC	(13) PRESC16													–			0.795	0.907	0.845	0.908	0.912
	(14) PRESC17													0.779	–		0.846		0.922		
	(15) PRESC19													0.726	0.792	–	0.804		0.859		

a All scores are computed based on polychoric correlation matrices for each subdimension. Shaded values indicate inter-item polychoric correlations of each factor.

The ICA’s outputs based on polychoric correlation matrices indicate that the ordinal alpha (*α*) values of all factors are above the 0.7 minimum value, and the sub-scales’ ordinal corrected item-total correlation coefficients are higher than the 0.20 limit score (Ravichandran and Rai, 1999; Jonsson, 2000; Streiner et al., 2003), according to findings in Table 4. Internal consistency was also evaluated using McDonald’s omega total (*ω_t_*) via a congeneric model based on the polychoric correlation matrix (McNeish, 2018). The scale demonstrated good reliability, indicating *ω_t_* = 0.922, 0.897, and 0.908, respectively (Flora, 2020). All items exhibited adequate standardized factor loadings (>0.45) on the primary construct (Brown, 2015; Tabachnick and Fidell, 2019). Additionally, every inter-item polychoric correlation exceeds the 0.30 cutoff. In CRA, every component CR omega total (*ω_t_*) score exceeds the specified limit value of 0.7 (Hayes and Coutts, 2020). Consequently, every reliability-related statistic shows how highly reliable the scale is. Given the previously mentioned results, it is feasible to conclude that the PRESC is valid and reliable.

## 6 Discussion and conclusion

The goal of the study was to create a validated Precariat Scale (PRESC), a 15-item measure that is quick to administer and complete. The PRESC framework, with factors structured around Sociocultural- economic (SCE), Job Insecurity, Uncertainty and Flexible Working (JIUFW), and Mental Condition (MC), provides a systematic approach to release people’s precarious structure. All statistical results of validity and reliability analyses show that the PRESC is a valid and reliable instrument for evaluating the precariat. Furthermore, the PRESC also has the benefit of evaluating structural and functional precariat at the same time. Since there are currently no Precariat Scales that measure the general adult population, the development of the PRESC will give academics and practitioners a useful instrument for assessing the precariat.

There are several restrictions on this study. The data used in this study are cross-sectional, and the results indicate the time period for data collection, because the data used in the analysis are acquired instantly. As a result, analysis conducted on data gathered at different periods may yield different conclusions. The online approach to data collection using social networks restricts our capacity to determine the proportion of respondents who choose not to complete the survey. Furthermore, the authors’ use of their own social networks, as well as the social networks of their colleagues employed by various academic and public institutions, universities, and pertinent student, alumni, and professional-based social network groups, to gather data online through social networks, may also be the cause of a biased sampling. Prior to drawing any conclusions, it is crucial to consider the features of our sample.

In this study, a scale was developed, and the validity and reliability were assessed regarding the so-called new dangerous class of the global precariat. The developed scale can be used for all genders, but to measure characteristics for females and their position within the precariat, the scale has to be developed by adding new gender-specific items during future research, and a femmecariat scale has to be developed as well.

## Data Availability

The original contributions presented in the study are included in the article/Supplementary material, further inquiries can be directed to the corresponding author.

## Author’s note

The study was developed by the authors from the dissertation supervised by Prof. Ahmet KESER (Ph.D.) and written by Müjgan ŞAHIN entitled “Deprem Sonrası Sosyal Durumun ve Göçün Kadının Sosyal-Kültürel Rolleri ve Dayanma Gücüne Etkisi: Prekarya İçinde Femmekarya’nın Yeni Rolü (The Impact of Post-Earthquake Social Conditions and Migration on Women’s Socio-Cultural Roles and Resilience: The New Role of Femmecariat within the Precariat)” at Hasan Kalyoncu University Post Graduate Institute, for the fulfillment of the Ph.D. Program at the Department of Political Science and IR., after being analyzed by Prof. Yunus GOKMEN during the conduct of the methodology of the study.
