# Supplementary material for: The Precariat Scale (PRESC): a psychometric tool for assessing precarity across mental, occupational, and sociocultural dimensions
Source: Front Psychol. 2026 Sep 1;17:1808999. doi: 10.3389/fpsyg.2026.1808999 (PMC13574830; doi:10.3389/fpsyg.2026.1808999)
Supplement: Supplementary file 1 [file Data_Sheet_1.PDF]

GeneralID	Sample ID	Gender	Age	Marital_Status	Education_Status
1	1	Male	51-55	Married	Graduate degree
2	2	Female	34-40	Single	Graduate degree
3	3	Female	29-33	Married	High School
4	4	Female	29-33	Single	Graduate degree
5	5	Male	17-22	Married	Graduate degree
6	6	Male	41-45	Single	Associate degree
7	7	Male	23-28	Single	Graduate degree
8	8	Male	29-33	Single	Bachelor’s degree
9	9	Male	17-22	Married	Bachelor’s degree
10	10	Male	17-22	Single	Bachelor’s degree
11	11	Male	17-22	Married	Bachelor’s degree
12	12	Female	17-22	Married	Bachelor’s degree
13	13	Male	23-28	Single	High School
14	14	Male	17-22	Married	Bachelor’s degree
15	15	Female	41-45	Single	Associate degree
16	16	Male	41-45	Married	High School
17	17	Female	41-45	Married	Bachelor’s degree
19	18	Female	56-60	Widowed	No education
20	19	Female	29-33	Single	Bachelor’s degree
21	20	Female	46-50	Married	Graduate degree
22	21	Female	34-40	Married	Graduate degree
23	22	Male	41-45	Single	Graduate degree
24	23	Male	34-40	Married	Bachelor’s degree
25	24	Female	17-22	Single	Graduate degree
26	25	Female	29-33	Married	Bachelor’s degree
28	26	Female	23-28	Single	Bachelor’s degree
29	27	Male	46-50	Married	Graduate degree
30	28	Male	51-55	Married	Bachelor’s degree
31	29	Female	34-40	Married	Primary/Secondary Sc
32	30	Male	23-28	Single	High School
33	31	Female	34-40	Married	Bachelor’s degree
34	32	Male	17-22	Married	High School
35	33	Female	23-28	Single	Bachelor’s degree
36	34	Male	56-60	Married	High School
37	35	Male	23-28	Single	Bachelor’s degree
39	36	Female	41-45	Single	High School
40	37	Male	46-50	Married	High School
41	38	Female	34-40	Married	Bachelor’s degree
42	39	Female	23-28	Single	Bachelor’s degree
43	40	Male	23-28	Single	Bachelor’s degree
44	41	Male	34-40	Married	Bachelor’s degree
45	42	Male	23-28	Single	Bachelor’s degree
46	43	Male	17-22	Single	High School
47	44	Male	61-65	Married	Primary/Secondary Sc
49	45	Female	29-33	Married	High School
50	46	Female	41-45	Single	Bachelor’s degree
51	47	Female	29-33	Married	High School
52	48	Male	46-50	Married	Primary/Secondary Sc
53	49	Female	41-45	Married	No education

**54**	**50**	Female	17-22	Married	Graduate degree
**55**	**51**	Female	41-45	Divorced/Separated	Primary/Secondary Sc
**57**	**52**	Male	29-33	Married	High School
**58**	**53**	Male	17-22	Married	Graduate degree
**59**	**54**	Male	46-50	Married	Bachelor’s degree
**60**	**55**	Female	29-33	Divorced/Separated	Bachelor’s degree
**61**	**56**	Male	23-28	Single	Bachelor’s degree
**62**	**57**	Female	34-40	Married	Bachelor’s degree
**63**	**58**	Female	29-33	Single	Graduate degree
**64**	**59**	Male	34-40	Single	Bachelor’s degree
**65**	**60**	Female	17-22	Single	Bachelor’s degree
**66**	**61**	Male	34-40	Married	Graduate degree
**68**	**62**	Female	29-33	Single	Bachelor’s degree
**69**	**63**	Female	29-33	Single	Bachelor’s degree
**71**	**64**	Female	41-45	Married	Bachelor’s degree
**72**	**65**	Male	17-22	Married	Bachelor’s degree
**73**	**66**	Female	23-28	Single	Bachelor’s degree
**74**	**67**	Female	46-50	Divorced/Separated	High School
**75**	**68**	Female	29-33	Single	Bachelor’s degree
**76**	**69**	Male	29-33	Single	Bachelor’s degree
**77**	**70**	Male	17-22	Married	Bachelor’s degree
**78**	**71**	Female	34-40	Married	High School
**79**	**72**	Female	17-22	Married	Bachelor’s degree
**80**	**73**	Male	17-22	Married	High School
**81**	**74**	Male	23-28	Single	Bachelor’s degree
**82**	**75**	Female	41-45	Single	Bachelor’s degree
**83**	**76**	Male	41-45	Single	Bachelor’s degree
**84**	**77**	Female	23-28	Married	Primary/Secondary Sc
**85**	**78**	Male	23-28	Married	Primary/Secondary Sc
**86**	**79**	Male	17-22	Married	Associate degree
**87**	**80**	Male	29-33	Single	Bachelor’s degree
**88**	**81**	Male	34-40	Married	Bachelor’s degree
**89**	**82**	Female	41-45	Divorced/Separated	High School
**90**	**83**	Female	41-45	Single	Bachelor’s degree
**91**	**84**	Female	29-33	Single	Associate degree
**92**	**85**	Female	23-28	Single	Bachelor’s degree
**93**	**86**	Female	34-40	Single	Graduate degree
**94**	**87**	Female	41-45	Single	Bachelor’s degree
**95**	**88**	Female	46-50	Married	Bachelor’s degree
**96**	**89**	Female	41-45	Single	Bachelor’s degree
**97**	**90**	Male	34-40	Married	Bachelor’s degree
**98**	**91**	Female	41-45	Single	Bachelor’s degree
**99**	**92**	Female	41-45	Divorced/Separated	Bachelor’s degree
**100**	**93**	Male	29-33	Married	Bachelor’s degree
**101**	**94**	Female	34-40	Single	Graduate degree
**102**	**95**	Female	46-50	Married	High School
**103**	**96**	Male	46-50	Married	High School
**104**	**97**	Male	17-22	Single	High School
**106**	**98**	Male	56-60	Married	High School
**107**	**99**	Female	41-45	Divorced/Separated	High School

**108**	**100**	Male	56-60	Married	Primary/Secondary Sc
**109**	**101**	Male	23-28	Single	Bachelor’s degree
**110**	**102**	Female	17-22	Single	High School
**112**	**103**	Female	17-22	Married	High School
**113**	**104**	Female	41-45	Single	Bachelor’s degree
**114**	**105**	Male	17-22	Single	High School
**115**	**106**	Female	34-40	Married	Associate degree
**116**	**107**	Female	29-33	Single	Graduate degree
**117**	**108**	Female	51-55	Married	Bachelor’s degree
**118**	**109**	Female	34-40	Single	Bachelor’s degree
**119**	**110**	Female	29-33	Single	Bachelor’s degree
**120**	**111**	Female	23-28	Single	Associate degree
**121**	**112**	Female	23-28	Married	Bachelor’s degree
**122**	**113**	Female	29-33	Single	Graduate degree
**123**	**114**	Male	41-45	Married	High School
**124**	**115**	Female	34-40	Married	Graduate degree
**125**	**116**	Female	23-28	Single	Bachelor’s degree
**126**	**117**	Female	23-28	Single	Bachelor’s degree
**127**	**118**	Female	23-28	Married	Bachelor’s degree
**128**	**119**	Male	41-45	Married	High School
**129**	**120**	Male	41-45	Married	High School
**130**	**121**	Female	46-50	Single	Bachelor’s degree
**131**	**122**	Female	41-45	Divorced/Separated	Associate degree
**132**	**123**	Male	23-28	Single	Bachelor’s degree
**133**	**124**	Female	23-28	Single	Bachelor’s degree
**134**	**125**	Female	29-33	Married	Bachelor’s degree
**135**	**126**	Male	34-40	Married	Graduate degree
**136**	**127**	Male	41-45	Married	Graduate degree
**137**	**128**	Female	34-40	Divorced/Separated	Primary/Secondary Sc
**138**	**129**	Female	41-45	Divorced/Separated	Primary/Secondary Sc
**140**	**130**	Female	41-45	Married	Primary/Secondary Sc
**141**	**131**	Male	23-28	Single	Bachelor’s degree
**142**	**132**	Male	29-33	Single	Bachelor’s degree
**143**	**133**	Female	41-45	Married	Primary/Secondary Sc
**144**	**134**	Male	29-33	Married	Bachelor’s degree
**145**	**135**	Female	23-28	Married	Bachelor’s degree
**146**	**136**	Female	51-55	Married	Primary/Secondary Sc
**147**	**137**	Male	23-28	Single	Associate degree
**149**	**138**	Female	29-33	Married	Bachelor’s degree
**150**	**139**	Male	23-28	Single	High School
**151**	**140**	Male	23-28	Single	Bachelor’s degree
**152**	**141**	Female	29-33	Married	Bachelor’s degree
**153**	**142**	Female	23-28	Single	Associate degree
**154**	**143**	Male	51-55	Married	Primary/Secondary Sc
**155**	**144**	Female	23-28	Single	Associate degree
**156**	**145**	Female	23-28	Single	Graduate degree
**157**	**146**	Male	23-28	Single	Bachelor’s degree
**158**	**147**	Female	41-45	Single	High School
**160**	**148**	Male	29-33	Single	Associate degree
**161**	**149**	Female	41-45	Single	Associate degree

**162**	**150**	Female	23-28	Single	Associate degree
**163**	**151**	Male	29-33	Married	Bachelor’s degree
**164**	**152**	Male	23-28	Single	Bachelor’s degree
**165**	**153**	Female	17-22	Single	Bachelor’s degree
**166**	**154**	Male	23-28	Single	Bachelor’s degree
**167**	**155**	Male	23-28	Single	Bachelor’s degree
**168**	**156**	Male	29-33	Married	Graduate degree
**169**	**157**	Male	41-45	Single	High School
**170**	**158**	Female	23-28	Married	Bachelor’s degree
**171**	**159**	Female	23-28	Single	Bachelor’s degree
**172**	**160**	Female	23-28	Single	Bachelor’s degree
**173**	**161**	Female	23-28	Single	Bachelor’s degree
**174**	**162**	Female	23-28	Single	Graduate degree
**175**	**163**	Female	41-45	Married	Associate degree
**176**	**164**	Female	23-28	Single	Bachelor’s degree
**177**	**165**	Male	29-33	Married	Primary/Secondary Sc
**178**	**166**	Female	41-45	Single	High School
**179**	**167**	Male	29-33	Single	Bachelor’s degree
**180**	**168**	Male	29-33	Single	High School
**181**	**169**	Female	29-33	Married	Graduate degree
**182**	**170**	Female	41-45	Married	Bachelor’s degree
**184**	**171**	Male	34-40	Married	High School
**185**	**172**	Male	29-33	Married	High School
**187**	**173**	Male	17-22	Married	Bachelor’s degree
**188**	**174**	Male	17-22	Married	Primary/Secondary Sc
**189**	**175**	Male	23-28	Married	Associate degree
**190**	**176**	Male	23-28	Single	Associate degree
**191**	**177**	Female	41-45	Married	Associate degree
**192**	**178**	Female	56-60	Divorced/Separated	Primary/Secondary Sc
**193**	**179**	Female	23-28	Single	Bachelor’s degree
**194**	**180**	Female	23-28	Single	Associate degree
**195**	**181**	Male	51-55	Married	Primary/Secondary Sc
**196**	**182**	Female	34-40	Single	Bachelor’s degree
**197**	**183**	Male	34-40	Married	High School
**199**	**184**	Male	41-45	Married	Bachelor’s degree
**200**	**185**	Female	51-55	Married	Bachelor’s degree
**201**	**186**	Male	41-45	Single	High School
**202**	**187**	Female	46-50	Married	High School
**203**	**188**	Male	34-40	Married	Bachelor’s degree
**204**	**189**	Male	34-40	Married	High School
**205**	**190**	Female	41-45	Married	Graduate degree
**206**	**191**	Male	29-33	Married	Primary/Secondary Sc
**207**	**192**	Female	41-45	Divorced/Separated	Primary/Secondary Sc
**208**	**193**	Female	34-40	Single	Bachelor’s degree
**209**	**194**	Female	29-33	Single	Associate degree
**210**	**195**	Male	23-28	Married	Primary/Secondary Sc
**211**	**196**	Female	23-28	Single	Bachelor’s degree
**212**	**197**	Female	34-40	Married	Associate degree
**213**	**198**	Male	29-33	Single	Bachelor’s degree
**214**	**199**	Male	17-22	Single	Associate degree

**216**	**200**	Female	17-22	Single	Bachelor’s degree
**217**	**201**	Female	23-28	Single	Bachelor’s degree
**218**	**202**	Male	34-40	Married	Bachelor’s degree
**219**	**203**	Female	23-28	Married	Associate degree
**220**	**204**	Male	41-45	Married	Bachelor’s degree
**221**	**205**	Female	41-45	Single	Bachelor’s degree
**222**	**206**	Female	46-50	Divorced/Separated	High School
**223**	**207**	Male	23-28	Single	Associate degree
**224**	**208**	Female	41-45	Married	Primary/Secondary Sc
**225**	**209**	Male	17-22	Married	High School
**226**	**210**	Male	17-22	Married	Graduate degree
**227**	**211**	Male	23-28	Single	Primary/Secondary Sc
**228**	**212**	Female	17-22	Married	High School
**229**	**213**	Male	46-50	Married	High School
**231**	**214**	Female	34-40	Married	Graduate degree
**232**	**215**	Female	41-45	Single	Associate degree
**233**	**216**	Female	17-22	Married	Bachelor’s degree
**234**	**217**	Female	29-33	Divorced/Separated	Bachelor’s degree
**235**	**218**	Female	23-28	Single	Bachelor’s degree
**236**	**219**	Female	29-33	Married	Associate degree
**237**	**220**	Male	46-50	Married	Bachelor’s degree
**238**	**221**	Female	46-50	Married	Primary/Secondary Sc
**239**	**222**	Female	34-40	Married	Bachelor’s degree
**240**	**223**	Female	61-65	Married	High School
**241**	**224**	Female	29-33	Married	Associate degree
**242**	**225**	Male	34-40	Married	High School
**243**	**226**	Female	34-40	Married	Primary/Secondary Sc
**244**	**227**	Male	41-45	Single	Graduate degree
**246**	**228**	Female	23-28	Single	Associate degree
**247**	**229**	Female	23-28	Single	Associate degree
**248**	**230**	Female	23-28	Single	High School
**249**	**231**	Female	29-33	Single	Graduate degree
**250**	**232**	Female	34-40	Married	Graduate degree
**251**	**233**	Male	34-40	Married	High School
**252**	**234**	Female	23-28	Married	Bachelor’s degree
**253**	**235**	Female	29-33	Married	Graduate degree
**254**	**236**	Male	29-33	Married	Bachelor’s degree
**255**	**237**	Female	34-40	Single	Bachelor’s degree
**256**	**238**	Female	17-22	Married	Graduate degree
**257**	**239**	Female	41-45	Single	Associate degree
**258**	**240**	Male	41-45	Married	Graduate degree
**259**	**241**	Male	34-40	Married	Graduate degree
**260**	**242**	Female	34-40	Widowed	High School
**261**	**243**	Male	29-33	Married	Bachelor’s degree
**262**	**244**	Male	46-50	Married	High School
**263**	**245**	Male	23-28	Single	High School
**264**	**246**	Male	29-33	Married	Bachelor’s degree
**265**	**247**	Female	46-50	Married	No education
**266**	**248**	Female	≥65	Married	No education
**267**	**249**	Female	23-28	Single	Bachelor’s degree

**268**	**250**	Female	23-28	Single	Associate degree
**269**	**251**	Male	34-40	Married	Associate degree
**270**	**252**	Female	46-50	Married	Bachelor’s degree
**271**	**253**	Male	29-33	Single	Graduate degree
**272**	**254**	Male	29-33	Single	Graduate degree
**273**	**255**	Female	41-45	Single	Bachelor’s degree
**274**	**256**	Female	34-40	Single	Bachelor’s degree
**275**	**257**	Male	23-28	Single	Bachelor’s degree
**277**	**258**	Female	34-40	Single	Bachelor’s degree
**278**	**259**	Female	29-33	Single	Bachelor’s degree
**279**	**260**	Female	34-40	Married	Bachelor’s degree
**280**	**261**	Male	34-40	Married	Bachelor’s degree
**281**	**262**	Male	34-40	Married	High School
**282**	**263**	Male	46-50	Married	Primary/Secondary Sc
**283**	**264**	Male	17-22	Married	Bachelor’s degree
**284**	**265**	Female	23-28	Single	Bachelor’s degree
**285**	**266**	Female	29-33	Divorced/Separated	Primary/Secondary Sc
**286**	**267**	Male	23-28	Single	Bachelor’s degree
**287**	**268**	Female	23-28	Single	Bachelor’s degree
**288**	**269**	Male	34-40	Married	Bachelor’s degree
**290**	**270**	Male	34-40	Married	High School
**291**	**271**	Female	23-28	Single	Bachelor’s degree
**292**	**272**	Male	23-28	Single	Bachelor’s degree
**293**	**273**	Male	23-28	Single	Bachelor’s degree
**294**	**274**	Male	41-45	Single	Bachelor’s degree
**295**	**275**	Male	34-40	Married	Bachelor’s degree
**296**	**276**	Female	23-28	Single	Graduate degree
**297**	**277**	Male	29-33	Married	Bachelor’s degree
**298**	**278**	Female	46-50	Married	Bachelor’s degree
**299**	**279**	Male	23-28	Single	Bachelor’s degree
**300**	**280**	Male	41-45	Single	High School
**301**	**281**	Female	29-33	Single	Bachelor’s degree
**302**	**282**	Male	41-45	Married	High School
**303**	**283**	Male	17-22	Married	Primary/Secondary Sc
**304**	**284**	Male	41-45	Divorced/Separated	High School
**305**	**285**	Female	34-40	Married	Primary/Secondary Sc
**306**	**286**	Female	23-28	Married	Bachelor’s degree
**308**	**287**	Female	23-28	Single	Associate degree
**309**	**288**	Female	23-28	Single	Bachelor’s degree
**310**	**289**	Male	41-45	Single	Associate degree
**311**	**290**	Female	29-33	Single	Bachelor’s degree
**312**	**291**	Male	41-45	Single	Associate degree
**313**	**292**	Female	23-28	Single	Bachelor’s degree
**314**	**293**	Male	41-45	Single	High School
**315**	**294**	Male	41-45	Single	Bachelor’s degree
**316**	**295**	Male	23-28	Single	Associate degree
**318**	**296**	Female	23-28	Married	Associate degree
**319**	**297**	Female	29-33	Married	High School
**320**	**298**	Male	46-50	Married	Primary/Secondary Sc
**321**	**299**	Female	17-22	Divorced/Separated	Graduate degree

**322**	**300**	Male	17-22	Married	Primary/Secondary Sc
**323**	**301**	Male	34-40	Married	Primary/Secondary Sc
**324**	**302**	Male	34-40	Married	High School
**325**	**303**	Male	46-50	Married	Primary/Secondary Sc
**326**	**304**	Female	29-33	Married	Bachelor’s degree
**327**	**305**	Male	41-45	Married	Primary/Secondary Sc
**328**	**306**	Male	17-22	Single	High School
**329**	**307**	Male	≥65	Married	Primary/Secondary Sc
**330**	**308**	Male	41-45	Single	High School
**331**	**309**	Female	34-40	Divorced/Separated	Primary/Secondary Sc
**332**	**310**	Male	≥65	Married	High School
**333**	**311**	Male	23-28	Single	Bachelor’s degree
**334**	**312**	Male	29-33	Married	Graduate degree
**335**	**313**	Male	41-45	Single	Bachelor’s degree
**336**	**314**	Female	61-65	Married	Primary/Secondary Sc
**337**	**315**	Male	34-40	Married	High School
**338**	**316**	Female	29-33	Single	High School
**339**	**317**	Male	51-55	Married	Bachelor’s degree
**340**	**318**	Female	41-45	Married	High School
**341**	**319**	Female	23-28	Single	Associate degree
**342**	**320**	Female	34-40	Married	No education
**343**	**321**	Male	34-40	Married	Primary/Secondary Sc
**344**	**322**	Female	41-45	Married	High School
**345**	**323**	Female	23-28	Married	Bachelor’s degree
**346**	**324**	Female	≥65	Widowed	No education
**347**	**325**	Female	34-40	Married	Primary/Secondary Sc
**348**	**326**	Male	23-28	Single	High School
**349**	**327**	Female	29-33	Single	Bachelor’s degree
**350**	**328**	Male	23-28	Single	Bachelor’s degree
**351**	**329**	Male	34-40	Married	High School
**352**	**330**	Male	29-33	Married	Associate degree
**353**	**331**	Male	29-33	Married	Bachelor’s degree
**354**	**332**	Female	41-45	Married	Bachelor’s degree
**355**	**333**	Female	41-45	Married	Bachelor’s degree
**356**	**334**	Male	41-45	Married	High School
**357**	**335**	Female	29-33	Married	Associate degree
**358**	**336**	Female	46-50	Married	Bachelor’s degree
**359**	**337**	Female	41-45	Single	Primary/Secondary Sc
**360**	**338**	Male	41-45	Single	High School
**361**	**339**	Female	41-45	Single	High School
**362**	**340**	Male	46-50	Married	Associate degree
**363**	**341**	Female	41-45	Married	Associate degree
**364**	**342**	Male	34-40	Divorced/Separated	High School
**365**	**343**	Male	29-33	Married	Bachelor’s degree
**366**	**344**	Female	41-45	Married	Primary/Secondary Sc
**367**	**345**	Female	41-45	Married	No education
**369**	**346**	Male	34-40	Single	Graduate degree
**370**	**347**	Male	34-40	Married	Associate degree
**371**	**348**	Male	23-28	Single	Graduate degree
**372**	**349**	Female	41-45	Single	Associate degree

**373**	**350**	Male	29-33	Married	Primary/Secondary Sc
**374**	**351**	Male	41-45	Married	Primary/Secondary Sc
**375**	**352**	Male	29-33	Married	Bachelor’s degree
**376**	**353**	Female	41-45	Married	Primary/Secondary Sc
**377**	**354**	Male	29-33	Single	Primary/Secondary Sc
**378**	**355**	Male	34-40	Single	Graduate degree
**379**	**356**	Female	34-40	Married	Bachelor’s degree
**380**	**357**	Male	41-45	Married	Bachelor’s degree
**381**	**358**	Male	56-60	Married	Bachelor’s degree
**382**	**359**	Female	29-33	Single	Bachelor’s degree
**383**	**360**	Male	23-28	Single	Associate degree
**384**	**361**	Male	34-40	Single	Bachelor’s degree
**385**	**362**	Female	29-33	Married	High School
**387**	**363**	Male	23-28	Married	Associate degree
**388**	**364**	Male	46-50	Married	High School
**389**	**365**	Male	51-55	Married	High School
**390**	**366**	Female	23-28	Married	Bachelor’s degree
**391**	**367**	Male	34-40	Single	Associate degree
**392**	**368**	Male	≥65	Married	Primary/Secondary Sc
**393**	**369**	Male	34-40	Married	Bachelor’s degree
**394**	**370**	Female	41-45	Single	Bachelor’s degree
**395**	**371**	Female	41-45	Married	Bachelor’s degree
**396**	**372**	Male	41-45	Married	Graduate degree
**397**	**373**	Male	56-60	Married	High School
**399**	**374**	Male	41-45	Divorced/Separated	Bachelor’s degree
**400**	**375**	Male	41-45	Single	Bachelor’s degree
**401**	**376**	Male	34-40	Married	Associate degree
**402**	**377**	Male	29-33	Married	Bachelor’s degree
**404**	**378**	Male	56-60	Married	Primary/Secondary Sc
**405**	**379**	Female	29-33	Married	High School
**406**	**380**	Female	29-33	Divorced/Separated	Primary/Secondary Sc
**407**	**381**	Male	29-33	Married	Bachelor’s degree
**408**	**382**	Male	29-33	Married	High School
**409**	**383**	Male	29-33	Married	High School
**410**	**384**	Male	41-45	Single	Associate degree
**411**	**385**	Male	41-45	Married	Bachelor’s degree
**412**	**386**	Male	29-33	Married	Associate degree
**413**	**387**	Female	29-33	Single	Graduate degree
**414**	**388**	Female	29-33	Married	Bachelor’s degree
**415**	**389**	Female	29-33	Single	Associate degree
**416**	**390**	Male	29-33	Single	Bachelor’s degree
**417**	**391**	Male	29-33	Single	Bachelor’s degree
**418**	**392**	Male	23-28	Married	Bachelor’s degree
**419**	**393**	Female	23-28	Married	High School
**421**	**394**	Male	34-40	Married	Primary/Secondary Sc

Occupation	Assurance_Status	PRESC1	PRESC2	PRESC3	PRESC5	PRESC6
Public sector	Not having social security	5	4	3	4	5
Public sector	Having social security	4	4	4	4	4
Self-employment	Not having social security	4	4	4	4	4
Private sector	Having social security	4	5	5	2	4
Public sector	Having social security	3	5	4	3	3
Private sector	Not having social security	2	2	5	2	4
Private sector	Having social security	4	4	3	3	3
Public sector	Having social security	3	3	5	5	5
Public sector	Having social security	3	2	2	2	4
Public sector	Having social security	2	1	1	1	3
Public sector	Having social security	5	2	2	2	4
Public sector	Having social security	2	2	2	2	2
Self-employment	Not having social security	4	4	4	4	4
Public sector	Having social security	4	4	4	4	4
Public sector	Having social security	2	2	2	1	4
Public sector	Having social security	3	3	3	3	3
Self-employment	Not having social security	2	2	2	2	4
Self-employment	Having social security	4	4	1	4	4
Self-employment	Not having social security	2	2	2	2	2
Public sector	Having social security	4	4	4	2	4
Public sector	Having social security	2	3	1	2	3
Self-employment	Not having social security	2	3	4	4	4
Public sector	Having social security	5	5	5	2	5
Public sector	Having social security	2	2	2	2	5
Public sector	Not having social security	1	4	3	3	3
Private sector	Having social security	4	4	5	5	5
Public sector	Having social security	2	2	4	4	4
Public sector	Having social security	4	2	2	2	4
Private sector	Not having social security	3	4	4	4	5
Private sector	Having social security	1	4	1	1	4
Public sector	Having social security	2	2	3	2	2
Private sector	Having social security	2	4	4	4	4
Private sector	Having social security	2	3	3	2	4
Private sector	Having social security	2	2	4	2	4
Private sector	Having social security	2	2	2	2	2
Private sector	Not having social security	1	4	2	2	5
Private sector	Not having social security	4	4	4	4	4
Public sector	Having social security	5	5	5	5	5
Self-employment	Not having social security	2	4	2	3	4
Public sector	Not having social security	2	2	4	2	4
Public sector	Having social security	3	4	4	3	4
Private sector	Having social security	2	2	2	2	2
Private sector	Having social security	3	4	3	3	4
Private sector	Having social security	5	5	3	5	3
Private sector	Having social security	4	4	4	3	4
Public sector	Having social security	4	4	4	3	4
Private sector	Having social security	2	4	5	4	4
Private sector	Having social security	4	4	3	4	4
Self-employment	Not having social security	3	4	3	3	3

Public sector	Having social security	5	5	5	4	5
Public sector	Not having social security	3	4	3	4	2
Private sector	Having social security	4	4	4	4	4
Public sector	Having social security	1	1	1	1	1
Self-employment	Having social security	2	3	3	3	3
Private sector	Having social security	2	4	2	2	4
Public sector	Having social security	3	1	2	3	3
Public sector	Having social security	3	4	4	2	4
Private sector	Having social security	2	3	2	2	5
Public sector	Having social security	2	3	2	2	2
Public sector	Having social security	2	3	3	2	4
Public sector	Having social security	2	2	2	2	3
Private sector	Having social security	2	2	2	2	3
Self-employment	Having social security	2	2	2	2	4
Private sector	Having social security	5	4	3	2	5
Public sector	Having social security	2	3	3	3	2
Public sector	Having social security	2	3	2	2	2
Private sector	Having social security	4	4	4	3	5
Self-employment	Having social security	2	4	3	2	4
Public sector	Having social security	4	4	4	4	4
Public sector	Having social security	3	3	3	3	5
Private sector	Not having social security	2	4	3	3	4
Public sector	Having social security	1	5	1	1	1
Private sector	Not having social security	2	2	2	2	2
Public sector	Having private social security	4	4	4	4	4
Private sector	Not having social security	3	2	2	3	4
Private sector	Having social security	1	1	1	1	1
Self-employment	Not having social security	3	4	4	3	1
Self-employment	Not having social security	3	4	2	2	3
Self-employment	Having social security	3	5	3	4	4
Public sector	Having social security	4	2	2	4	4
Public sector	Having social security	4	3	3	3	3
Public sector	Having social security	2	2	2	2	2
Self-employment	Not having social security	3	3	3	3	3
Public sector	Having social security	2	2	4	4	4
Private sector	Having social security	3	4	4	1	1
Public sector	Having social security	3	3	3	3	4
Public sector	Having social security	4	1	2	1	1
Public sector	Having social security	2	2	2	2	2
Private sector	Having social security	2	4	2	2	2
Public sector	Having social security	2	4	3	3	3
Private sector	Not having social security	3	4	4	3	4
Public sector	Having social security	2	2	2	2	2
Public sector	Having social security	2	1	4	3	3
Private sector	Having social security	2	2	3	1	2
Self-employment	Having private social security	4	4	3	3	4
Private sector	Not having social security	4	3	4	4	4
Public sector	Not having social security	4	4	4	4	4
Private sector	Not having social security	3	5	3	3	5
Self-employment	Having private social security	2	4	2	2	4

Private sector	Not having social security	3	3	3	3	4
Private sector	Not having social security	4	5	4	4	4
Public sector	Having social security	5	4	5	4	4
Private sector	Having social security	2	2	2	5	5
Self-employment	Having social security	2	1	1	1	3
Self-employment	Not having social security	2	2	1	2	2
Private sector	Not having social security	3	4	4	4	3
Public sector	Having social security	3	3	4	3	1
Self-employment	Having social security	4	5	5	5	5
Self-employment	Having social security	4	4	4	4	4
Public sector	Having social security	4	2	2	3	4
Private sector	Having social security	4	5	3	5	5
Private sector	Having social security	3	3	3	3	3
Private sector	Having private social security	2	2	4	3	3
Private sector	Having social security	2	5	5	5	5
Private sector	Having social security	2	5	4	2	2
Private sector	Not having social security	2	2	3	2	3
Self-employment	Having social security	1	1	2	2	2
Private sector	Not having social security	5	5	5	5	5
Private sector	Having social security	4	2	2	3	4
Private sector	Having social security	1	1	1	4	4
Public sector	Having social security	4	4	4	4	4
Self-employment	Not having social security	2	2	2	2	2
Self-employment	Having private social security	2	4	4	4	4
Self-employment	Not having social security	5	5	5	5	5
Self-employment	Having social security	1	2	1	1	2
Public sector	Having social security	3	2	2	1	3
Public sector	Having social security	2	4	4	2	3
Public sector	Not having social security	4	4	2	4	2
Public sector	Not having social security	3	4	4	2	2
Public sector	Having social security	3	3	3	1	4
Private sector	Not having social security	4	4	4	3	4
Private sector	Having social security	4	5	5	3	3
Public sector	Having social security	1	1	1	1	1
Public sector	Having social security	2	3	2	2	3
Private sector	Having social security	4	5	4	4	5
Public sector	Having social security	1	2	4	4	4
Private sector	Having social security	3	4	4	4	4
Public sector	Having social security	4	4	4	4	4
Private sector	Not having social security	4	4	4	4	4
Public sector	Having social security	1	1	1	1	2
Private sector	Not having social security	4	4	5	1	1
Public sector	Having social security	4	4	4	4	2
Self-employment	Having social security	4	2	4	4	4
Public sector	Having social security	2	3	4	2	5
Self-employment	Having private social security	1	1	1	1	1
Private sector	Not having social security	1	2	4	4	4
Private sector	Not having social security	4	4	3	4	4
Self-employment	Not having social security	4	4	4	4	1
Private sector	Having social security	2	4	2	2	2

Public sector	Having social security	2	3	3	3	3
Public sector	Having social security	4	4	4	4	4
Public sector	Having social security	3	3	2	3	2
Private sector	Not having social security	4	4	4	4	4
Private sector	Having social security	2	4	2	1	2
Private sector	Having social security	1	2	1	1	1
Private sector	Having social security	1	1	1	2	1
Private sector	Having private social security	1	1	1	1	1
Private sector	Having social security	1	1	1	1	1
Private sector	Not having social security	1	1	1	1	1
Private sector	Having social security	2	1	1	2	1
Private sector	Not having social security	4	2	3	4	4
Private sector	Having social security	1	1	2	1	1
Private sector	Having social security	2	4	4	2	4
Private sector	Having social security	1	2	2	1	4
Private sector	Having social security	3	4	4	2	4
Private sector	Not having social security	3	4	4	1	4
Public sector	Having social security	2	2	4	2	2
Private sector	Having social security	3	1	1	3	4
Public sector	Having social security	1	1	1	1	1
Public sector	Having social security	4	5	4	4	4
Public sector	Having social security	4	3	2	1	3
Self-employment	Having social security	4	4	2	2	4
Public sector	Having social security	2	2	2	2	2
Private sector	Not having social security	4	3	3	4	4
Private sector	Having social security	1	2	2	1	1
Private sector	Not having social security	1	1	1	1	1
Private sector	Having social security	1	1	1	1	1
Private sector	Not having social security	1	1	1	1	1
Private sector	Having social security	1	1	1	1	1
Private sector	Having social security	1	1	1	1	1
Self-employment	Having social security	1	1	1	1	1
Public sector	Having social security	1	4	4	1	4
Public sector	Having private social security	3	3	3	3	5
Public sector	Having social security	1	1	4	1	3
Public sector	Having social security	2	4	2	4	2
Self-employment	Having private social security	1	1	1	1	1
Public sector	Having social security	3	3	3	3	4
Public sector	Having private social security	2	2	2	2	4
Public sector	Having private social security	2	2	2	3	3
Public sector	Having social security	2	2	2	3	3
Private sector	Not having social security	2	2	2	3	3
Self-employment	Having social security	2	2	1	2	2
Public sector	Having social security	2	4	4	3	4
Private sector	Having social security	4	4	4	2	4
Private sector	Not having social security	2	2	4	2	2
Self-employment	Having social security	2	4	3	3	3
Public sector	Having social security	2	2	4	2	2
Self-employment	Not having social security	1	1	1	1	1
Self-employment	Having private social security	4	3	3	3	4

Public sector	Having social security	1	3	3	3	3
Public sector	Having social security	1	1	3	1	1
Public sector	Having social security	3	4	4	4	4
Self-employment	Not having social security	5	5	1	3	5
Private sector	Having social security	4	4	3	1	2
Private sector	Not having social security	3	4	4	3	3
Private sector	Not having social security	2	4	4	1	3
Self-employment	Having social security	2	5	2	2	2
Private sector	Not having social security	3	4	4	3	3
Public sector	Having social security	3	4	5	3	3
Private sector	Having social security	1	1	1	1	1
Private sector	Having private social security	2	2	2	2	2
Private sector	Having social security	1	2	4	3	5
Self-employment	Having social security	5	4	5	5	5
Public sector	Having social security	4	4	4	4	4
Self-employment	Not having social security	2	1	1	1	4
Private sector	Not having social security	4	3	4	1	4
Private sector	Having social security	4	4	3	4	4
Private sector	Having social security	1	1	1	1	1
Private sector	Having social security	1	1	1	1	1
Private sector	Having private social security	1	2	3	2	2
Self-employment	Having social security	3	3	4	3	3
Private sector	Having social security	4	4	2	4	4
Self-employment	Having social security	2	2	4	3	4
Private sector	Having social security	4	4	4	3	2
Private sector	Having social security	4	4	4	3	5
Self-employment	Not having social security	2	2	2	2	2
Private sector	Having social security	2	2	1	1	2
Private sector	Having social security	2	2	2	2	2
Private sector	Having social security	1	2	1	2	2
Self-employment	Not having social security	1	4	2	2	4
Self-employment	Having social security	2	2	3	2	3
Public sector	Having social security	1	1	1	1	2
Public sector	Having private social security	4	5	4	4	4
Public sector	Having social security	3	4	4	2	4
Public sector	Having social security	4	5	5	4	5
Public sector	Having social security	1	1	1	1	1
Private sector	Having social security	3	4	4	3	4
Public sector	Having social security	2	2	4	1	3
Self-employment	Having social security	1	1	2	2	2
Public sector	Having social security	2	4	5	2	4
Self-employment	Having social security	5	5	1	5	5
Private sector	Not having social security	1	1	1	1	1
Self-employment	Having social security	1	3	3	2	3
Private sector	Having social security	1	4	1	1	3
Self-employment	Not having social security	1	3	1	1	2
Public sector	Having social security	1	2	2	2	2
Self-employment	Not having social security	2	4	4	1	1
Self-employment	Not having social security	4	4	4	4	4
Private sector	Having social security	1	4	1	1	1

Public sector	Having social security	2	2	4	1	1
Private sector	Having social security	3	4	4	3	4
Public sector	Having social security	3	4	3	3	3
Self-employment	Having social security	3	4	3	3	3
Self-employment	Not having social security	1	3	2	2	2
Private sector	Not having social security	1	4	3	1	2
Public sector	Having social security	1	1	1	1	1
Self-employment	Not having social security	4	4	4	4	4
Public sector	Having social security	1	1	1	1	1
Public sector	Having social security	2	2	2	2	2
Self-employment	Not having social security	2	2	2	2	4
Public sector	Having social security	1	5	1	2	2
Private sector	Having social security	2	4	2	2	2
Public sector	Having social security	1	1	1	1	1
Public sector	Having social security	1	1	1	1	1
Public sector	Having social security	4	4	4	4	4
Public sector	Having social security	1	1	1	1	1
Public sector	Having social security	4	1	4	3	4
Private sector	Having social security	5	5	5	5	5
Private sector	Having social security	1	2	2	2	2
Self-employment	Not having social security	5	5	5	5	5
Private sector	Not having social security	2	2	2	2	2
Private sector	Having social security	1	1	1	1	1
Self-employment	Having private social security	2	4	4	1	2
Private sector	Not having social security	1	2	3	1	2
Private sector	Having social security	1	1	1	1	1
Public sector	Having social security	4	5	4	3	5
Public sector	Having social security	4	1	2	4	3
Public sector	Having social security	4	4	4	4	4
Private sector	Not having social security	1	4	1	1	3
Private sector	Having social security	1	4	5	3	3
Self-employment	Having social security	4	1	4	4	3
Private sector	Having social security	3	3	3	3	3
Public sector	Having social security	4	4	5	2	3
Self-employment	Not having social security	2	2	1	1	1
Self-employment	Having social security	1	2	1	1	1
Private sector	Having social security	4	4	4	3	4
Private sector	Having social security	3	4	4	4	5
Public sector	Not having social security	2	4	4	4	4
Public sector	Having private social security	4	5	5	4	4
Self-employment	Having private social security	1	2	2	2	3
Public sector	Having social security	2	2	2	2	3
Public sector	Having private social security	1	4	1	1	1
Private sector	Having social security	2	1	1	3	2
Self-employment	Not having social security	3	4	3	3	4
Public sector	Having social security	1	1	1	1	3
Private sector	Having social security	4	4	4	3	4
Self-employment	Not having social security	2	2	2	2	2
Private sector	Having social security	1	1	3	2	2
Public sector	Having social security	1	1	2	1	1

Private sector	Having social security	3	3	3	2	2
Private sector	Having social security	2	1	1	1	4
Private sector	Having social security	1	4	3	3	3
Self-employment	Having social security	2	4	4	3	4
Private sector	Having social security	4	4	4	4	4
Private sector	Having social security	2	1	1	1	2
Private sector	Having social security	2	3	1	1	3
Self-employment	Having social security	2	3	5	5	3
Private sector	Not having social security	2	3	4	3	3
Public sector	Not having social security	3	4	4	4	4
Private sector	Having social security	1	5	2	2	2
Public sector	Having social security	4	4	4	4	4
Public sector	Not having social security	2	2	2	2	3
Private sector	Having social security	1	1	1	1	5
Self-employment	Having social security	3	4	3	4	4
Private sector	Having social security	1	2	2	1	2
Private sector	Having social security	1	4	4	1	3
Public sector	Having social security	4	4	1	2	3
Public sector	Having social security	3	3	3	5	4
Public sector	Having social security	1	4	1	1	3
Private sector	Not having social security	2	2	2	3	3
Private sector	Having social security	3	3	2	2	4
Self-employment	Not having social security	5	5	5	4	4
Public sector	Having social security	4	5	5	5	5
Public sector	Having social security	4	2	2	4	4
Public sector	Not having social security	2	4	2	2	2
Self-employment	Not having social security	4	5	4	4	5
Private sector	Not having social security	1	4	3	2	4
Self-employment	Not having social security	2	2	2	2	2
Public sector	Having social security	1	4	4	2	3
Private sector	Having social security	2	3	2	4	2
Public sector	Having social security	2	1	3	3	4
Public sector	Having social security	4	4	4	3	4
Public sector	Having social security	3	4	3	3	3
Private sector	Having social security	3	3	2	2	4
Private sector	Not having social security	5	4	4	3	5
Public sector	Having social security	2	2	4	4	2
Private sector	Not having social security	3	2	2	4	1
Private sector	Not having social security	3	3	3	3	3
Private sector	Not having social security	4	4	4	4	4
Self-employment	Having social security	3	3	3	3	3
Private sector	Not having social security	2	2	2	2	4
Private sector	Having social security	2	1	2	2	3
Public sector	Having social security	4	4	4	4	4
Self-employment	Having social security	3	3	3	3	3
Self-employment	Not having social security	2	4	4	4	4
Private sector	Not having social security	4	3	2	3	4
Public sector	Having social security	5	2	2	1	4
Self-employment	Not having social security	4	3	2	4	4
Private sector	Having social security	1	1	1	1	4

Private sector	Not having social security	1	1	1	1	1
Self-employment	Having social security	1	1	1	1	1
Public sector	Having social security	1	1	1	1	4
Private sector	Not having social security	1	1	1	1	1
Public sector	Having social security	2	2	2	2	3
Public sector	Having social security	1	5	5	1	1
Public sector	Having social security	2	4	2	3	3
Public sector	Having social security	2	4	2	4	4
Public sector	Having social security	3	3	3	3	3
Public sector	Having social security	2	4	2	2	2
Private sector	Having social security	1	5	4	4	4
Public sector	Having social security	2	2	2	4	4
Private sector	Having social security	2	3	4	2	4
Private sector	Having social security	1	4	3	1	3
Public sector	Having social security	3	3	4	4	5
Self-employment	Having social security	2	2	2	2	2
Private sector	Having social security	2	4	4	4	4
Public sector	Having social security	3	3	4	3	3
Self-employment	Having social security	2	4	2	3	4
Self-employment	Having social security	4	2	4	3	4
Public sector	Having social security	2	4	4	3	4
Public sector	Having social security	3	4	3	3	4
Self-employment	Having social security	2	4	2	2	4
Self-employment	Having social security	4	4	4	3	4
Self-employment	Having social security	2	4	4	4	4
Private sector	Having social security	3	4	4	4	4
Public sector	Having social security	1	2	1	3	3
Public sector	Having social security	4	4	4	4	4
Self-employment	Having social security	1	1	1	1	1
Public sector	Having social security	1	2	2	3	3
Public sector	Not having social security	5	5	2	4	5
Public sector	Having social security	2	4	3	1	4
Private sector	Having social security	4	5	4	3	4
Private sector	Having social security	4	3	4	3	4
Private sector	Having social security	1	2	1	1	1
Self-employment	Having social security	4	4	4	4	4
Public sector	Having social security	5	5	5	3	4
Private sector	Having social security	1	2	2	2	2
Self-employment	Not having social security	2	4	4	5	4
Private sector	Having social security	3	4	2	4	4
Public sector	Having social security	4	4	2	3	2
Private sector	Having social security	4	1	2	4	4
Self-employment	Having social security	1	2	1	1	4
Public sector	Having social security	2	4	2	2	4
Self-employment	Having social security	3	3	3	3	3

PRESC7	PRESC8	PRESC11	PRESC12	PRESC13	PRESC14	PRESC15	PRESC16	PRESC17
5	5	2	2	5	4	2	2	2
4	5	3	4	5	4	2	5	4
4	4	2	4	4	2	2	3	2
2	5	2	5	4	2	4	3	2
3	4	2	4	4	2	4	5	5
2	2	2	4	4	2	2	3	2
3	2	2	2	3	2	2	5	5
5	2	1	2	2	1	1	1	1
2	4	2	2	3	2	2	4	4
1	3	2	2	4	4	3	5	5
2	2	4	3	5	3	2	3	3
2	2	4	3	3	1	1	4	2
4	4	4	2	4	4	4	3	3
4	4	4	3	1	3	2	5	4
2	3	4	2	4	2	2	3	2
3	3	5	3	5	3	3	5	4
2	4	2	1	4	2	2	2	2
4	4	4	5	4	5	5	1	1
3	4	3	4	4	4	4	2	2
4	4	2	2	1	2	2	2	2
2	5	4	4	4	2	2	4	4
5	5	4	3	2	2	2	4	3
4	4	4	4	3	5	3	4	4
5	4	4	2	4	2	2	2	4
4	4	3	1	2	1	2	4	4
4	2	4	5	5	4	5	2	3
2	2	5	5	5	2	4	5	5
2	2	5	2	4	5	5	3	2
4	4	4	5	5	2	2	3	3
1	1	5	3	5	3	3	5	5
2	2	2	2	2	2	2	3	2
4	4	3	4	3	2	2	5	5
2	2	3	3	4	2	3	4	4
4	4	2	4	5	2	2	5	4
2	1	2	3	3	3	2	4	3
5	5	1	4	4	2	4	2	1
4	4	4	5	2	4	4	2	2
5	5	4	4	2	4	4	4	5
2	4	3	4	5	4	5	5	5
4	4	2	4	4	4	2	3	2
3	4	4	3	5	4	2	5	2
1	2	1	1	1	2	2	2	2
4	4	2	2	2	2	2	2	2
5	5	2	1	1	1	1	1	1
4	4	2	2	2	2	2	2	2
4	4	2	2	3	4	4	3	3
4	4	2	2	2	2	2	2	2
4	4	4	5	4	2	3	3	4
3	3	3	4	4	4	4	5	4

5	5	1	2	1	1	2	5	3
4	4	1	3	3	4	4	5	1
4	4	3	4	4	2	2	3	4
1	1	5	5	5	5	5	5	5
3	3	3	3	3	3	3	3	3
2	4	3	3	5	4	4	4	4
4	2	4	4	4	5	5	3	2
4	2	2	2	2	3	3	3	2
5	4	2	5	4	2	2	3	2
2	1	3	4	4	4	4	4	4
2	4	2	3	3	3	2	4	2
2	2	5	5	3	5	5	5	5
1	1	3	5	5	3	3	5	5
2	1	3	5	4	4	4	5	5
4	4	3	4	2	2	2	1	1
1	4	2	2	2	3	3	2	5
3	1	4	5	5	5	5	5	5
5	4	3	3	3	2	2	3	4
2	2	2	3	3	2	2	4	4
4	4	2	3	3	2	2	2	2
2	4	5	5	5	3	3	1	1
2	3	2	5	3	4	5	4	5
1	1	3	5	3	3	4	5	5
2	2	4	3	4	3	4	4	4
4	4	5	5	5	3	2	5	5
4	2	3	4	3	2	2	3	2
1	1	5	5	3	5	5	5	4
4	4	4	4	4	4	4	5	5
3	2	4	4	4	4	3	4	3
5	3	2	5	2	3	4	5	5
2	4	2	4	4	2	2	2	2
3	2	2	2	3	3	5	3	4
2	3	2	2	2	3	3	2	2
3	4	3	5	2	4	4	5	4
4	3	2	4	5	3	2	3	3
3	4	5	5	5	5	5	3	3
2	4	3	2	2	3	3	3	4
1	1	5	4	5	4	4	4	5
2	4	5	3	5	5	5	5	5
2	2	5	5	5	4	3	4	4
4	3	4	4	4	4	4	5	3
3	4	3	2	5	2	2	2	2
4	2	4	4	2	4	4	2	2
3	4	2	2	4	2	4	5	5
1	4	4	3	3	3	4	4	4
4	4	2	5	2	3	5	5	5
4	4	4	2	2	2	1	1	1
4	4	4	2	4	2	2	4	4
3	4	4	5	5	4	5	5	5
2	1	3	4	4	3	4	2	3

3	2	5	5	5	5	3	5	5
4	1	2	5	5	3	2	1	1
4	4	4	3	4	2	2	2	2
4	5	2	1	2	1	2	2	2
1	1	4	4	5	5	5	5	5
2	1	2	1	1	1	1	3	3
4	4	3	4	2	2	5	3	2
3	1	3	4	5	2	2	5	3
5	5	1	1	1	1	1	1	1
4	4	2	2	2	2	2	2	2
2	4	2	2	3	4	4	3	2
5	5	3	5	4	4	2	2	1
3	3	3	2	3	4	3	3	2
4	4	4	3	3	2	3	1	1
1	1	1	1	1	2	2	5	1
2	4	4	4	1	2	2	4	4
2	2	5	5	3	3	3	5	4
2	2	3	4	3	3	3	3	3
5	5	2	3	3	1	4	1	1
4	4	2	4	5	2	2	3	3
4	2	2	2	3	2	2	5	5
4	4	1	1	1	2	1	1	1
2	2	3	3	5	4	5	5	5
4	4	2	4	2	2	2	2	4
5	5	4	4	3	3	2	1	1
1	1	4	5	5	4	5	1	1
3	3	4	5	5	3	4	5	5
3	2	3	4	4	4	4	5	4
3	4	4	4	4	4	2	3	3
2	2	4	4	4	4	4	4	4
3	1	2	5	3	2	4	3	3
4	4	2	3	4	2	2	4	3
4	4	5	2	5	2	1	4	4
1	1	1	2	2	2	1	1	5
2	2	5	5	2	4	4	4	4
5	5	3	4	2	3	2	1	1
4	4	4	4	4	3	4	4	4
4	4	2	2	2	2	3	4	4
4	4	4	4	4	4	4	2	2
4	4	4	4	4	4	4	4	4
2	2	2	2	2	2	2	4	4
1	2	4	5	5	4	5	5	2
2	4	1	2	2	2	2	2	2
4	4	5	3	4	5	5	5	5
3	4	4	5	3	4	4	4	3
1	2	3	3	4	5	4	5	5
4	4	2	2	1	2	2	4	4
4	4	2	2	4	3	3	3	2
1	4	4	5	2	4	4	4	2
3	2	2	4	2	4	2	4	2

3	2	2	2	2	2	2	1	1
4	4	3	2	2	1	1	2	2
2	3	5	3	5	5	3	5	4
4	4	1	1	1	1	1	2	2
1	2	5	5	5	5	5	3	2
1	2	5	5	5	5	5	5	5
1	2	4	5	5	5	5	5	5
1	2	4	5	5	5	5	5	4
1	1	5	5	5	5	5	5	5
1	1	5	5	5	5	5	5	5
2	2	5	5	5	5	5	5	5
4	4	2	5	2	3	1	5	5
1	1	1	5	5	2	5	5	5
2	4	3	4	4	3	3	2	2
3	2	5	4	4	4	4	3	4
2	1	2	2	4	2	2	3	4
5	4	4	3	4	2	2	2	2
2	2	4	4	4	4	2	4	4
2	3	4	5	5	2	2	5	5
1	1	5	5	5	5	5	5	2
4	4	2	1	2	2	2	2	2
4	4	5	5	5	5	5	4	2
3	4	2	4	2	3	2	4	3
2	2	3	4	5	2	2	3	3
4	4	2	2	3	3	2	2	2
1	1	4	5	5	4	4	5	5
1	1	5	5	5	5	4	5	5
1	1	5	5	5	5	5	5	5
1	1	5	5	5	5	5	5	5
1	1	5	5	5	5	5	5	5
1	1	5	5	5	5	5	5	4
1	1	5	5	5	5	5	4	4
4	4	4	4	4	4	4	4	4
3	3	1	1	3	3	2	2	2
1	3	3	4	3	2	2	3	2
2	4	4	5	4	5	5	3	4
1	1	5	5	5	5	2	3	3
5	5	3	5	5	3	3	4	4
2	3	2	2	2	4	2	2	4
2	3	5	5	5	4	3	5	4
3	3	2	4	4	4	3	5	4
2	2	4	4	4	4	5	4	4
2	1	1	5	5	5	5	5	5
3	2	2	4	3	2	2	2	2
4	4	1	1	1	1	1	1	1
2	2	2	4	4	4	4	4	2
3	1	2	3	4	3	4	4	2
2	2	4	4	5	3	2	3	3
1	1	3	3	3	2	2	4	4
4	4	3	4	4	3	5	5	3

3	3	3	3	3	3	3	3	3
1	1	4	4	4	4	2	4	4
4	4	1	2	1	1	1	1	1
2	1	2	4	5	4	4	3	4
3	2	4	4	3	2	3	4	4
2	2	4	4	4	2	2	3	2
1	1	3	5	4	3	5	3	2
2	4	5	5	3	3	5	5	5
3	4	4	5	5	5	4	5	5
3	4	2	4	3	3	3	3	3
1	1	1	5	5	2	3	3	1
2	2	4	4	2	4	4	4	4
5	2	2	5	5	2	2	5	5
5	4	2	2	2	1	1	2	2
4	4	4	2	5	2	1	2	1
4	2	4	4	4	2	2	3	3
4	4	5	5	5	5	5	5	4
4	4	3	4	5	3	2	4	5
1	2	2	5	5	5	5	4	4
1	1	5	5	5	5	5	4	3
1	1	5	5	5	5	2	5	4
4	4	3	2	5	2	3	3	1
4	3	2	2	3	2	2	3	2
2	2	5	4	4	2	2	2	2
2	4	3	4	4	2	3	4	2
5	5	2	3	2	2	3	3	1
2	1	4	5	4	4	5	5	5
1	2	4	5	4	5	5	5	3
2	1	4	5	5	4	4	4	4
1	1	5	5	5	4	5	5	5
5	4	3	4	3	3	3	4	4
2	2	5	5	5	4	5	5	5
1	1	3	3	4	4	5	5	4
4	4	4	5	4	3	4	2	3
3	2	4	3	4	4	3	5	3
4	4	5	5	5	2	2	4	5
1	1	1	1	1	1	1	1	1
4	4	3	5	4	4	4	5	4
4	3	4	3	3	3	3	3	3
2	2	4	4	5	3	2	5	2
2	4	3	4	4	3	3	4	1
1	5	2	2	2	2	2	1	1
3	1	4	4	4	4	4	4	1
2	2	4	5	5	3	4	3	5
1	1	5	5	5	3	4	4	3
1	1	2	4	4	4	4	4	5
2	2	5	5	4	4	3	5	4
1	1	5	5	3	2	5	5	5
4	4	4	2	4	4	2	3	3
1	1	5	5	3	2	1	5	5

1	1	2	2	3	2	1	2	1
4	4	2	5	2	2	2	4	3
3	3	4	5	5	4	4	4	2
3	2	3	5	3	3	3	4	4
2	3	3	4	5	4	2	4	2
3	2	4	2	4	4	4	4	2
3	3	1	4	4	4	4	4	4
4	3	2	1	1	2	2	2	2
1	1	5	5	5	5	5	5	3
3	5	3	4	3	4	1	4	4
4	2	4	2	2	4	4	4	4
3	1	1	1	1	2	2	1	1
3	4	4	4	4	3	3	4	4
1	1	5	5	5	5	5	5	5
1	1	5	5	5	5	5	5	5
4	4	4	2	2	2	2	2	2
1	1	1	5	5	5	5	5	5
4	4	2	2	1	1	2	4	4
5	5	5	1	1	1	5	1	1
1	2	3	4	5	3	2	4	4
5	5	3	5	5	5	5	5	1
2	1	5	5	5	5	3	5	5
1	1	5	5	5	5	5	5	5
2	1	2	2	1	2	2	5	5
1	2	2	4	4	5	5	5	5
1	3	2	2	5	2	2	5	5
4	3	3	3	3	3	3	3	3
4	1	1	1	1	2	1	5	5
4	4	3	2	1	1	1	1	1
1	4	5	4	5	4	4	2	3
3	3	3	3	5	3	3	5	5
5	4	3	5	5	2	3	5	5
3	4	3	3	3	3	3	3	3
4	4	5	5	5	4	4	3	2
1	1	4	4	2	2	5	5	5
1	1	5	5	3	5	5	1	5
4	4	2	4	4	3	3	2	2
3	5	2	1	2	2	1	2	2
4	4	5	5	5	4	2	1	1
4	4	2	2	2	2	2	2	2
4	4	5	4	5	4	4	5	5
2	1	5	4	5	2	3	3	3
3	1	5	5	3	4	5	5	5
2	2	1	1	1	2	1	1	1
5	4	5	5	4	3	3	1	1
1	2	3	5	4	2	4	5	5
4	4	2	2	2	2	2	2	2
2	2	2	4	4	2	2	2	2
2	1	1	2	1	1	1	2	2
1	4	4	5	5	5	5	5	5

3	5	2	1	1	1	1	2	3
2	4	3	3	4	4	4	4	4
2	4	4	5	4	5	2	2	2
4	3	3	4	3	3	4	4	2
4	4	4	4	4	2	4	2	2
1	1	4	3	4	5	5	5	4
3	3	4	4	4	2	4	3	3
2	2	5	5	5	4	4	5	1
2	4	4	4	3	3	4	5	4
1	5	2	5	5	3	4	5	5
2	3	3	5	5	5	5	5	5
4	4	2	2	2	3	3	3	2
2	2	3	3	3	2	2	1	1
1	5	1	1	1	1	1	1	4
4	4	2	4	4	2	2	2	2
1	2	2	5	5	4	2	2	2
5	5	1	5	5	5	5	5	3
4	3	2	3	3	3	3	2	3
4	1	5	5	5	5	5	5	5
1	1	4	4	4	5	4	5	4
3	4	5	3	5	4	3	4	5
4	4	5	2	5	3	5	5	4
4	4	1	4	3	3	1	2	1
5	5	1	1	1	1	1	1	1
2	4	4	4	4	4	4	2	2
2	4	2	4	3	4	3	2	2
5	5	4	4	5	4	4	4	2
3	4	4	3	4	3	2	4	4
2	3	3	2	3	3	3	1	1
4	2	3	3	3	4	4	3	5
3	3	3	3	3	3	3	3	4
3	4	3	5	5	4	3	3	3
5	5	2	4	3	3	3	3	3
5	3	2	3	2	4	4	3	3
3	4	5	5	4	4	5	3	2
5	5	2	2	1	2	2	5	1
2	3	3	5	3	4	2	3	4
2	5	2	3	3	4	3	2	4
3	4	2	3	3	4	4	4	3
4	4	4	4	4	4	4	2	2
3	3	3	3	4	4	4	4	4
4	4	2	2	2	2	2	2	2
3	1	1	2	1	1	1	1	2
4	4	2	4	2	2	2	2	2
4	4	3	4	4	3	3	2	2
4	4	4	4	4	3	3	3	2
4	4	1	3	3	2	2	4	2
2	1	2	1	1	1	2	1	1
4	4	2	5	5	3	4	4	2
2	2	5	5	3	4	5	5	5

1	1	5	5	5	5	5	5	5
1	1	5	5	5	5	5	5	3
2	4	2	5	5	4	5	4	3
1	1	5	3	5	3	5	5	5
1	1	5	3	5	5	3	4	5
1	1	1	3	2	3	3	1	1
2	3	4	3	3	4	4	4	3
3	4	1	1	2	1	2	2	2
2	3	3	2	3	2	2	3	2
3	3	3	4	4	4	4	4	4
4	2	4	3	3	4	4	5	5
4	2	3	2	2	4	2	4	2
4	4	4	3	3	4	4	2	2
1	4	4	4	4	3	4	4	3
5	5	1	1	2	1	2	1	1
2	2	2	4	4	4	3	5	5
4	2	5	5	5	5	5	5	5
4	3	4	3	5	3	3	5	5
4	1	3	5	5	4	4	5	5
4	4	4	4	4	3	3	3	3
4	4	2	1	2	2	5	3	4
3	4	2	2	3	2	2	4	4
2	4	2	2	3	2	3	3	2
2	2	4	3	4	2	2	2	2
4	4	4	4	4	4	4	4	4
4	4	5	5	5	4	4	5	1
2	4	5	5	4	5	5	5	4
4	4	4	2	2	4	2	2	2
1	3	5	5	5	5	3	5	5
4	4	4	4	5	4	4	4	2
5	5	2	1	1	1	1	3	2
1	1	1	2	2	1	1	3	2
4	4	2	2	2	1	1	3	4
4	4	2	3	2	2	3	3	3
1	1	5	5	3	5	5	5	5
4	5	2	2	2	3	3	1	1
3	4	5	2	5	2	2	2	1
2	2	5	4	4	5	5	5	1
2	4	2	2	4	2	2	2	2
4	4	3	3	3	3	4	3	1
4	5	2	4	2	2	1	2	2
4	4	2	4	4	3	3	4	2
1	4	5	5	5	5	5	5	4
3	3	2	2	3	2	2	4	4
3	2	3	4	4	3	4	4	3

PRESC19
2
4
2
4
5
4
3
1
4
5
3
5
2
4
3
3
4
1
2
4
5
4
5
3
1
2
5
1
2
5
4
5
4
4
2
1
2
5
5
2
5
2
2
1
2
2
3
3
4

1
1
3
5
3
3
2
2
1
5
4
5
5
5
2
2
5
4
4
3
1
3
5
4
5
2
3
5
3
5
2
3
2
3
4
3
4
2
5
4
4
2
2
4
3
5
1
4
3
2

3
1
2
2
5
3
1
3
1
2
2
4
3
1
1
1
5
3
2
4
4
1
5
4
1
1
5
4
2
4
2
3
4
3
5
1
3
4
2
2
4
2
2
5
2
5
2
2
2
4

2
1
3
2
2
5
5
5
5
5
5
5
5
2
4
2
2
4
5
1
1
3
4
2
2
4
5
5
5
5
3
4
4
2
1
2
2
3
3
4
5
3
5
2
1
3
3
2
4
3

3
3
1
4
4
2
1
5
5
2
1
4
3
2
1
3
5
1
3
3
4
1
4
2
2
1
5
5
4
5
4
3
5
2
3
4
1
3
3
4
2
1
4
3
4
5
3
5
4
5

2
4
4
4
2
2
3
2
5
4
3
1
4
5
5
2
5
3
1
5
1
5
5
5
5
5
3
3
4
5
5
5
3
2
5
5
2
2
4
2
3
4
5
1
1
5
2
2
2
5

4
2
3
2
2
2
3
3
3
5
5
2
1
4
2
2
4
3
5
5
3
4
1
1
2
2
4
4
1
3
4
3
5
2
2
1
4
2
3
2
4
2
3
2
3
2
1
1
1
5

5
5
2
5
3
1
4
2
2
4
5
3
3
3
1
5
5
2
5
3
1
3
1
2
4
5
3
2
5
4
2
2
3
2
2
1
1
1
2
4
2
4
4
4
3
