# Supplementary material for: The Precariat Scale (PRESC): a psychometric tool for assessing precarity across mental, occupational, and sociocultural dimensions
Source: Front Psychol. 2026 Sep 1;17:1808999. doi: 10.3389/fpsyg.2026.1808999 (PMC13574830; doi:10.3389/fpsyg.2026.1808999)
Supplement: Supplementary file 2 [file Supplementary_file_1.docx]

**Appendix A:** The Results of Analyses for Pilot Study

**Table A1.** Results of Item Analysis of PRESC

| **Item**  **ID*** | **Ordinal corrected item-total correlation** | **Ordinal Alpha if-item-deleted** | **Ordinal Alpha** |
| --- | --- | --- | --- |
| PRESC1 | 0.719 | 0.854 | 0.871 |
| PRESC2 | 0.466 | 0.865 |  |
| PRESC3 | 0.610 | 0.859 |  |
| PRESC5 | 0.567 | 0.861 |  |
| PRESC6 | 0.637 | 0.858 |  |
| PRESC7 | 0.513 | 0.863 |  |
| PRESC8 | 0.563 | 0.861 |  |
| ***PRESC9*** | ***0.178*** | ***0.877*** |  |
| PRESC11** | 0.329 | 0.871 |  |
| PRESC12** | 0.455 | 0.866 |  |
| PRESC13** | 0.492 | 0.864 |  |
| PRESC14** | 0.617 | 0.859 |  |
| PRESC15** | 0.384 | 0.869 |  |
| PRESC16** | 0.607 | 0.859 |  |
| PRESC17** | 0.690 | 0.856 |  |
| PRESC19** | 0.625 | 0.858 |  |
| ***PRESC20***** | ***0.027*** | ***0.883*** |  |

n=78. (*): Items 4, 10, and 18 were removed before item analysis. (**): Reverse scores are computed. Bold values indicate items with ordinal-corrected item-total correlations below the 0.2 threshold.

**Table A2.** The Results of EFA and CA of the Pilot Study

| **Items** | **EFA** | | | | | | | | **CA (****Inter-Item Polychoric Correlations)** | | | | | | | | | | | | | | | | | | | | | | | | | | | | |
| --- | --- | --- | --- | --- | --- | --- | --- | --- | --- | --- | --- | --- | --- | --- | --- | --- | --- | --- | --- | --- | --- | --- | --- | --- | --- | --- | --- | --- | --- | --- | --- | --- | --- | --- | --- | --- | --- |
|  | **Factor Loadings** | | | | | **Total Variance Explained (%)** | | **(1)** | | | **(2)** | | **(3)** | | **(4)** | | **(5)** | | **(6)** | | **(7)** | | **(8)** | | **(9)** | | **(10)** | | **(11)** | | **(12)** | | **(13)** | | **(14)** | | **(15)** |
|  | **SCE** | | **JIUFW** | | **MC** |  |  |  |  |  |  |  |  |  |  |  |  |  |  |  |  |  |  |  |  |  |  |  |  |  |  |  |  |  |  |  |  |
| **(1)** PRESC1 | **0.896** | 0.093 | | -0.009 | | 62.928 | **-** | | |  | |  | |  | |  | |  | |  | |  | |  | |  | |  | |  | |  | |  | |  | |
| **(2)** PRESC2 | **0.670** | -0.112 | | 0.087 | |  | 0.587 | | | **-** | |  | |  | |  | |  | |  | |  | |  | |  | |  | |  | |  | |  | |  | |
| **(3)** PRESC3 | **0.748** | 0.137 | | -0.053 | |  | 0.647 | | | 0.717 | | **-** | |  | |  | |  | |  | |  | |  | |  | |  | |  | |  | |  | |  | |
| **(4)** PRESC5 | **0.720** | -0.003 | | 0.037 | |  | 0.758 | | | 0.460 | | 0.539 | | **-** | |  | |  | |  | |  | |  | |  | |  | |  | |  | |  | |  | |
| **(5)** PRESC6 | **0.796** | -0.065 | | 0.111 | |  | 0.741 | | | 0.450 | | 0.566 | | 0.600 | | **-** | |  | |  | |  | |  | |  | |  | |  | |  | |  | |  | |
| **(6)** PRESC7 | **0.839** | -0.121 | | -0.023 | |  | 0.690 | | | 0.522 | | 0.548 | | 0.595 | | 0.804 | | **-** | |  | |  | |  | |  | |  | |  | |  | |  | |  | |
| **(7)** PRESC8 | **0.813** | 0.096 | | -0.129 | |  | 0.755 | | | 0.513 | | 0.593 | | 0.549 | | 0.664 | | 0.602 | | **-** | |  | |  | |  | |  | |  | |  | |  | |  | |
| **(8)** PRESC11 | -0.046 | **0.607** | | -0.018 | |  |  | | |  | |  | |  | |  | |  | |  | | - | |  | |  | |  | |  | |  | |  | |  | |
| **(9)** PRESC12 | -0.003 | **0.692** | | 0.014 | |  |  | | |  | |  | |  | |  | |  | |  | | 0.388 | | - | |  | |  | |  | |  | |  | |  | |
| **(10)** PRESC13 | 0.087 | **0.841** | | -0.131 | |  |  | | |  | |  | |  | |  | |  | |  | | 0.451 | | 0.619 | | - | |  | |  | |  | |  | |  | |
| **(11)** PRESC14 | 0.077 | **0.670** | | 0.174 | |  |  | | |  | |  | |  | |  | |  | |  | | 0.459 | | 0.514 | | 0.581 | | - | |  | |  | |  | |  | |
| **(12)** PRESC15 | -0.077 | **0.622** | | 0.089 | |  |  | | |  | |  | |  | |  | |  | |  | | 0.462 | | 0.413 | | 0.438 | | 0.589 | | - | |  | |  | |  | |
| **(13)** PRESC16 | -0.079 | 0.140 | | **0.832** | |  |  | | |  | |  | |  | |  | |  | |  | |  | |  | |  | |  | |  | | - | |  | |  | |
| **(14)** PRESC17 | 0.071 | 0.043 | | **0.876** | |  |  | | |  | |  | |  | |  | |  | |  | |  | |  | |  | |  | |  | | 0.817 | | - | |  | |
| **(15)** PRESC19 | 0.030 | -0.059 | | **0.916** | |  |  | | |  | |  | |  | |  | |  | |  | |  | |  | |  | |  | |  | | 0.765 | | 0.851 | | - | |

n=78. Bold values indicate the dominant factor loadings for each item.

**Table A3.** The Findings of the Ordinal Corrected Item-Total Correlations of Two Subsamples

|  | **Subsample-1**  (n_1_=39) | **Subsample-2**  (n_2_=39) |
| --- | --- | --- |
| PRESC1 | 0.750 | 0.712 |
| PRESC2 | 0.508 | 0.481 |
| PRESC3 | 0.640 | 0.632 |
| PRESC5 | 0.669 | 0.712 |
| PRESC6 | 0.797 | 0.731 |
| PRESC7 | 0.595 | 0.542 |
| PRESC8 | 0.525 | 0.558 |
| PRESC11* | 0.509 | 0.462 |
| PRESC12* | 0.508 | 0.483 |
| PRESC13* | 0.592 | 0.574 |
| PRESC14* | 0.690 | 0.627 |
| PRESC15* | 0.520 | 0.503 |
| PRESC16* | 0.557 | 0.583 |
| PRESC17* | 0.680 | 0.710 |
| PRESC19* | 0.621 | 0.607 |

(*): Reverse scores are computed.
