# Supplementary material for: The Precariat Scale (PRESC): a psychometric tool for assessing precarity across mental, occupational, and sociocultural dimensions
Source: Front Psychol. 2026 Sep 1;17:1808999. doi: 10.3389/fpsyg.2026.1808999 (PMC13574830; doi:10.3389/fpsyg.2026.1808999)
Supplement: Supplementary file 3 [file Supplementary_file_2.docx]

**Appendix B:** Prekaryalaşma Ölçeği Geliştirme Soruları **(Scale Development Questionnaire on the Precariat)**

**Demografik Sorular (Lütfen demografik yapı sorularında size en uygun gelen seçeneği işaretleyiniz)**

[Demographic Questions (Please select the option that best describes your demographic status)]

1. **Cinsiyet (Gender):**

a. Kadın (**Female**)	b. Erkek (**Male**)	c. Belirtmek istemiyorum (**I prefer not to disclose**)

2. **Medeni durumunuzu en iyi tanımlayan ifade hangisidir (Which statement best describes your marital status)?**
   a. Evli (**Married**
   b. Bekar (**Single)**
   c. Dul (**Widowed**)
   d. Boşandı/Ayrı yaşıyor (**Divorced/Separated**)
3. **Çalışıyorsa çalıştığı kurum veya işyeri durumu (Occupation):**
a. Kamu (**Public sector**)
b. Özel Sektör (**Private sector**)
c. Kendi İşi (**Self-employment**)
4. **Sosyal güvenceye sahip olma durumu (Status of having assurance / social security due to primary employment):**

a.	Özel sosyal güvenlik sistemine sahip olmak (**Having private social security**)

b.	Sosyal güvenlik sistemine sahip olmak (**Having social security**)

c.	Sosyal güvenlik sistemine sahip olmamak (**Not having social security**)

5. **Yaş (Age):**

a. 18-22	b. 23-28	c. 29-33	d. 34-40	e. 41-45

f. 46-50	g. 51-55	h. 56-60	i. 61-65

6. **Eğitim Durumu (Education Status):**
   a. Okuryazar değil (**No education**)
   b. İlköğretim (İlkokul/Ortaokul) (**Primary/Secondary school**)
   c. Lise (**High school**)
   d. Ön lisans (2 yıllık meslek yüksekokulu) (**Associate degree**)
   e. Lisans (Üniversite 4 ve üstü yıllık fakülte) (**Bachelor’s degree**)
   f. Lisansüstü (Yüksek lisans/Doktora) (**Graduate degree**)

**PRESC-Prekaryalaşma Ölçeği: Prekarya Olma Durumu Göstergelerine İlişkin Ölçek**

**(PRESC-Precariat Scale: A Scale Related to Indicators of Being in the Precariat)**

**Lütfen aşağıdaki soruları ölçeğe göre doldurunuz (Please answer the following questions according to the scale)**

| **5** | **4** | **3** | **2** | **1** |
| --- | --- | --- | --- | --- |
| Kesinlikle Katılıyorum  (**Strongly Agree**) | Katılıyorum  (**Agree**) | Kısmen Katılıyorum  (**Partially Agree**) | Katılmıyorum  (**Disagree**) | Kesinlikle Katılmıyorum  (**Strongly Disagree**) |

| **Sorular (Questions)** | **5** | **4** | **3** | **2** | **1** |
| --- | --- | --- | --- | --- | --- |
| **1.** Her yaz tatile giderim (**I go on vacation every summer**). |  |  |  |  |  |
| **2.** Haftada en az bir gün arkadaşlarımla vakit geçiririm (**I spend time with my friends at least one day a week**). |  |  |  |  |  |
| **3.** Bir hobiye sahibim ve zaman ayırabiliyorum (**I have a hobby and can devote time to it**). |  |  |  |  |  |
| **5.** Kazancımı yeterli buluyorum (**I find my income sufficient**). |  |  |  |  |  |
| **6.** Temel besin maddelerine ulaşabiliyorum (**I am able to access basic food supplies**). |  |  |  |  |  |
| **7.** Yaşadığım evden memnunum ve yeterli alana sahibim (**I am satisfied with my home and have enough space**). |  |  |  |  |  |
| **8.** Sağlık giderlerimi karşılayabiliyorum ve sağlık sigortam var (**I can cover my health expenses and have health insurance**). |  |  |  |  |  |
| **11.** Yaptığım işte yükselme şansım yok (**There is no chance for promotion in my job**). |  |  |  |  |  |
| **12.** Güvencesiz bir işte olmak beni kaygılandırıyor (**Being in insecure employment makes me anxious**). |  |  |  |  |  |
| **13.** İşten ayrılırsam kolaylıkla iş bulamam (**If I lose my job, I would have difficulty finding a new one**). |  |  |  |  |  |
| **14.** İşimde dinlenme zamanım neredeyse hiç yok (**I almost never have time to rest at work**). |  |  |  |  |  |
| **15.** İş saatlerimin düzensizliğinden dolayı zamanı yönetemiyorum (**I cannot manage my time due to** **irregular working hours**). |  |  |  |  |  |
| **16.** Psikolojik olarak tükenmiş hissediyorum (**I feel psychologically burned out**). |  |  |  |  |  |
| **17.** Kendimi yalnız hissediyorum (**I feel lonely**). |  |  |  |  |  |
| **19.** Psikolojik bir desteğe ihtiyacım var (**I need psychological support**). |  |  |  |  |  |

**Note 1**: Items 4, 10, and 18 were entirely removed from the scale because the experts deemed them redundant or outside the scope of the target construct, and items 9 and 20, whose ordinal corrected item-total correlations were below the 0.2 threshold, were eliminated in the pilot study during the validation study. **Finally, a 15-item scale was developed, but the question numbers were not changed during the study.**

**Note 2:** The precariat scale development and validity study were conducted in Turkish. The English interpretation of the items is also provided in brackets but **researchers who prefer to use the English version have to conduct a cultural validity and adaptation study before their final research in English**.

**Factor 1: SCE /** Sosyokültürel ve ekonomik faktör / **Sociocultural-economic factor** (**1-2-3-5-6-7-8^th^ questions**).

**Factor 2: JIUFW /** İş Güvencesizliği, Belirsizlik ve Esnek Çalışma faktörü / **Job Insecurity, Uncertainty and Flexible Working factor** (**11-12-13-14-15^th^ questions**)

**Factor 3: MC /** Zihinsel Sağlık / Ruh Sağlığı Durumu faktörü **/ Mental Condition (16-17-19^th^ questions).**
